# Disruption of the pentraxin 3/CD44 interaction as an efficient therapy for triple‐negative breast cancers

**DOI:** 10.1002/ctm2.724

**Published:** 2022-01-28

**Authors:** Yu‐Wei Hsiao, Jhih‐Ying Chi, Chien‐Feng Li, Lei‐Yi Chen, Yi‐Ting Chen, Hsin‐Yin Liang, Yu‐Chih Lo, Jhen‐Yi Hong, Chin‐Pin Chuu, Liang‐Yi Hung, Jyun‐Yi Du, Wen‐Chang Chang, Ju‐Ming Wang

**Affiliations:** ^1^ Department of Biotechnology and Bioindustry Sciences, College of Bioscience and Biotechnology National Cheng Kung University Tainan Taiwan R. O. C.; ^2^ Department of Pathology Chi‐Mei Medical Center Tainan Taiwan R. O. C.; ^3^ Institute of Cellular and System Medicine National Health Research Institutes Miaoli County Taiwan R. O. C.; ^4^ Graduate Institute of Medical Sciences, College of Medicine Taipei Medical University Taipei Taiwan R. O. C.; ^5^ International Research Center for Wound Repair and Regeneration National Cheng Kung University Tainan Taiwan R. O. C.; ^6^ Department of Physiology, College of Medicine National Cheng Kung University Tainan Taiwan R. O. C.; ^7^ Graduate Institute of Medicine, College of Medicine Kaohsiung Medical University Kaohsiung Taiwan R. O. C.

**Keywords:** CD44 and tumour microenvironment, NF‐κB, pentraxin 3 (PTX3), triple‐negative breast cancer cells (TNBC)

## Abstract

Due to the heterogeneity and high frequency of genome mutations in cancer cells, targeting vital protumour factors found in stromal cells in the tumour microenvironment may represent an ideal strategy in cancer therapy. However, the regulation and mechanisms of potential targetable therapeutic candidates need to be investigated. An in vivo study demonstrated that loss of pentraxin 3 (PTX3) in stromal cells significantly decreased the metastasis and growth of cancer cells. Clinically, our results indicate that stromal PTX3 expression correlates with adverse prognostic features and is associated with worse survival outcomes in triple‐negative breast cancer (TNBC). We also found that transforming growth factor beta 1 (TGF‐β1) induces PTX3 expression by activating the transcription factor CCAAT/enhancer binding protein delta (CEBPD) in stromal fibroblasts. Following PTX3 stimulation, CD44, a PTX3 receptor, activates the downstream ERK1/2, AKT and NF‐κB pathways to specifically contribute to the metastasis/invasion and stemness of TNBC MDA‐MB‐231 cells. Two types of PTX3 inhibitors were developed to disrupt the PTX3/CD44 interaction and they showed a significant effect on attenuating growth and restricting the metastasis/invasion of MDA‐MB‐231 cells, suggesting that targeting the PTX3/CD44 interaction could be a new strategy for future TNBC therapies.

## Graphical abstract

**Graphical Highlights**

1. TGF‐β1 stimulates *PTX3* transcription by activating the transcription factor CEBPD in breast CAFs.
2. The PTX3/CD44 interaction contributes to the metastasis/invasion and stemness of MDA‐MB‐231 via TWIST2, MMP2, RANKL and NANOG in response to AKT‐ and ERK1/2‐activated NF‐κB.
3. PTX3 antibodies and synthetic peptides can disrupt the PTX3/CD44 interaction and attenuate and restrict the stemness and metastasis/invasion of TNBC.

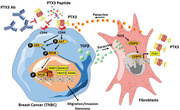

## 1 INTRODUCTION

Tumour microenvironment (TME) has been suggested to engage in cancer initiation and promotion of tumour development. Triple‐negative breast cancer (TNBC) is characterised by aggressive and highly recurrent lesions. Importantly, a correlation between activated stroma and highly metastatic tumours and poor prognosis has been demonstrated in TNBC patients.^1^, ^2^, ^3^ Clinically, the therapeutic options for TNBC treatment are quite limited and inefficient. Surgery combined with chemotherapy and radiotherapy is the standard strategy for treating TNBC owing to its lack of response to hormonal therapies such as tamoxifen, aromatase inhibitors and targeted therapies such as HER2 inhibitors. Therefore, it is urgent to develop an effective targeted therapy for controlling metastatic TNBC.

The TME stromal cells have been suggested to support cancer progression.^4^ Detected cancer‐associated fibroblasts (CAFs) are predominantly myofibroblasts,^5^ and they respond differently to various microenvironment signals, exhibiting distinct functions. Clinical and epidemiological evidence has demonstrated a strong association between the density of CAFs and poor prognosis in many cancers.^6^, ^7^, ^8^, ^9^ An increasing number of studies has indicated that activated stroma is positively correlated with highly metastatic tumours and poor outcome in TNBC patients.^10^ In addition, due to the heterogeneity and high frequency of genome mutations in cancer cells, targeting protein molecules in cancer cells tends to result in intrinsic drug resistance and is consequently associated with therapeutic failure. However, the efficacy of targeting vital protumour factors produced by CAFs, blocking the interactions between cancer cells and the surrounding cells, remains unclear. Therefore, identifying vital protumour factors and assessing the impact of inhibiting them as an effective targeted therapy to control metastatic TNBC merits investigation.

In advanced breast cancer, transforming growth factor beta 1 (TGF‐β1) has been suggested to promote tumour progression by modulating the epithelial‐mesenchymal transition of cancer cells and promoting invasion, migration and metastasis.^11^, ^12^, ^13^ Recent studies have revealed that TGF‐β1 promotes tumour stem‐like properties in breast cancers and leads to the expansion of chemotherapy‐resistant populations and tumour recurrence.^14^, ^15^ However, details related to the extrinsic protumour effects in response to TGF‐β1 and their mediation, especially regarding the communication between cancer cells and CAFs, remain largely unknown.

As a type I transmembrane glycoprotein, CD44 can be expressed by many various cell types, including macrophages, neutrophils, and T and B cells. Several ligands, including fibronectin, osteopontin and collagen, have been suggested to interact with CD44, but the best known CD44 ligand is hyaluronan (HA). In cancer biology, CD44 shows a significant positive correlation with tumour recurrence, mortality, metastasis and invasion in malignant cancers.^16^, ^17^ Various alternative splicing of CD44 isoforms is aberrantly expressed in breast tumours, including TNBC, and has been suggested to be involved in the metastatic process and stemness ability.^17^, ^18^, ^19^ Therefore, identifying and inhibiting an ideal and specific CD44 interactor involved in the CD44‐mediated protumour role might provide a solution and new options for advanced breast cancer therapy.

In addition to serving as a multifunctional soluble receptor, several studies suggest that pentraxin 3 (PTX3) can be a secretory factor that is suggested to play functional roles in recognising microbial moieties, exhibits opsonic activity, regulates the complement cascade and inflammation and is involved in tissue remodelling.^20^, ^21^, ^22^ Epigenetic regulation makes an important impact on the regulation of *PTX3* transcription in cancer cells.^23^, ^24^ Additionally, we previously identified *PTX3* transcription in response to inflammatory factors in stromal cells and demonstrated the involvement of PTX3 in the exacerbation of inflammation‐associated diseases.^25^, ^26^, ^27^ Except for being a potent tumour suppressor,^24^, ^28^, ^29^ increasing evidence shows that an abundance of PTX3 is associated with either the grade of malignancy or a poor prognosis and contributes to acquired chemoresistance and cancer metastasis/invasion.^30^, ^31^, ^32^, ^33^, ^34^ However, regarding PTX3 function as a protumour regulator, several issues require clarification, such as its receptor(s), committed downstream signalling and therapeutic potential, especially in patients for whom drug treatments and therapy are not advised.

## 2 RESULTS

### 2.1 Stromal *PTX3* expression correlates with adverse prognostic features and is associated with poor outcomes in TNBC

**FIGURE 1:**
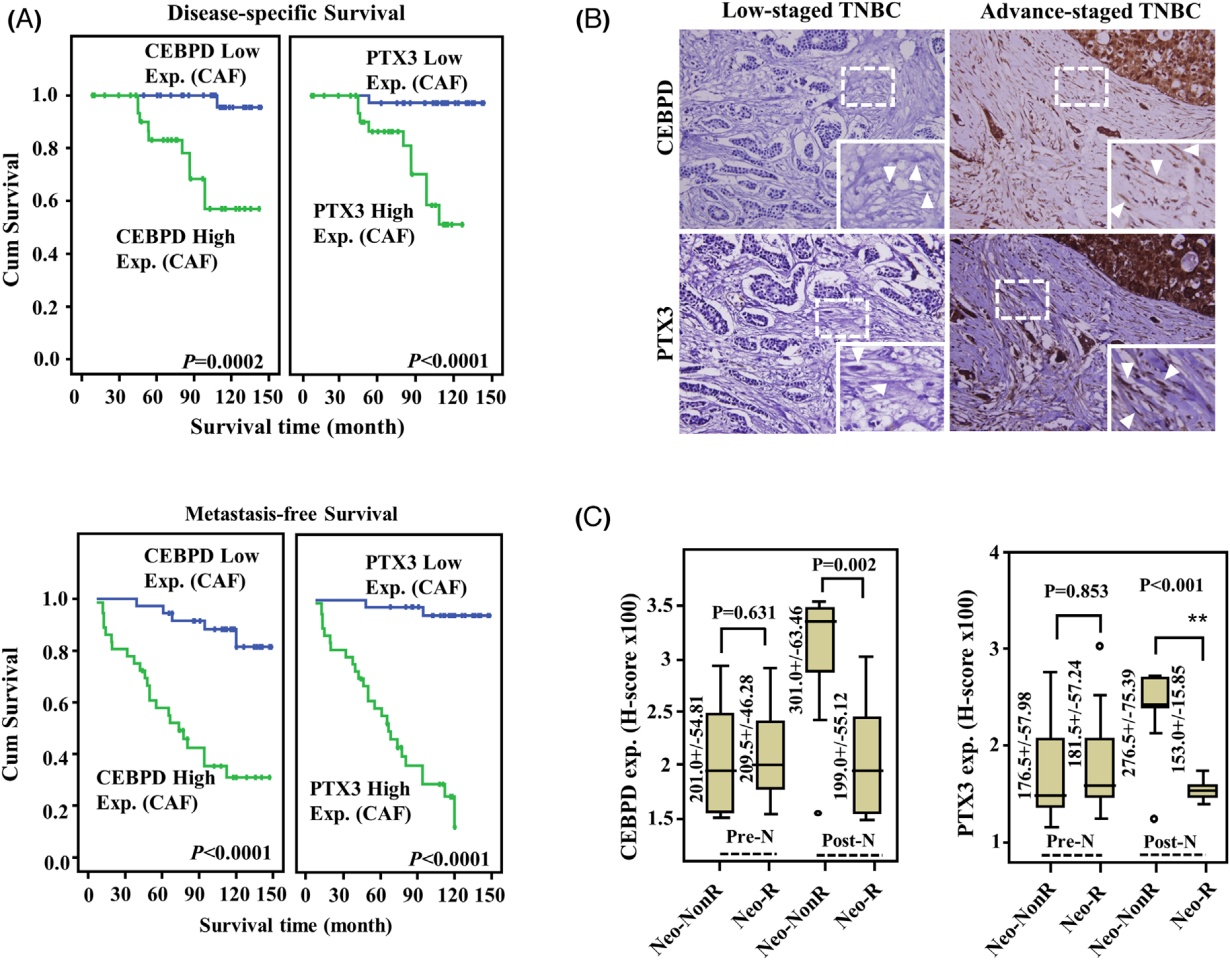
Stromal pentraxin 3 (PTX3) expression is positively correlates with worse outcomes in triple‐negative breast cancer (TNBC) patients. (A) In primary breast cancers, higher CCAAT/enhancer binding protein delta (CEBPD) and PTX3 levels in cancer‐associated fibroblasts (CAFs) are associated with reduced disease‐specific and metastasis‐free survival times. (B) An immunohistochemistry assay was performed in TNBC patients. The expression of CEBPD and PTX3 in the CAFs in representative TNBC in early (*left* panel) and advanced (*right* panel) stages. The tumour is surrounded by spindle‐shaped fibroblasts (as indicated by the arrow) in advanced‐stage TNBC. (C) In those receiving neoadjuvant chemotherapy, CAFs CEBPD and PTX3 expression levels are significantly increased in non‐responders. Abbreviations: ANOVA, Analysis of variance; C/T, chemotherapy; H.R., Hazard ratio; KO, knock out; Pre‐N, pre‐neoadjuvant C/T biopsy samples; Post‐N, post‐neoadjuvant C/T biopsy samples

The transcription factor CCAAT/enhancer binding protein delta (CEBPD) activates the *PTX3* gene in stromal cells, contributing to cancer and chemoresistant tumour progression.^25^, ^30^ However, CEBPD‐associated PTX3 regulation has not been examined in detail regarding its clinical or epidemiological characteristics in various cancers, including breast cancer. Here, we assessed the association between TNBC progression and stromal CEBPD and PTX3 expression in TNBC specimens. We found that higher levels of CEBPD and PTX3 in the CAFs of the TME were significantly and positively associated with nodal metastasis (*p* = .006 and <.001, respectively) and poor disease‐specific survival (DSS, *p* = .0002 and <.0001, respectively) and metastasis‐free survival (MeFS, *p* < .0001 and <.0001, respectively) rates in 72 TNBC cases. Moreover, CEBPD and PTX3 expression was significantly correlated with the prognosis in multivariate analysis of MeFS (*p* = .035 and .012, respectively; Figure 1A,B, Tables 1, 2, 3). Notably, stromal CEBPD and PTX3 expression were highly increased in TNBC resistant to adjuvant chemotherapy (non‐responders) (*p* = .002 and <.001, respectively; Figure 1C).

**TABLE 1:** Univariate log‐rank analysis for disease‐specific survival (DSS) and metastasis‐free survival (MeFS) in 72 triple‐negative breast cancer (TNBC) patients

			DSS		MeFS	
Parameters	Category	No. of case	No. of event	*p*‐Value	No. of event	*p*‐Value
Age (years)	<60	57	7	.0942	22	.5347
	≧60	15	4		6	
Primary tumour (T)	T1	29	2	.0507	3	<.0001*
	T2	34	7		17	
	T3–T4	9	2		8	
Nodal status (N)	N0	47	3	.0002*	10	<.0001*
	N1–N2	25	8		18	
Stage	I	24	1	<.0001*	1	<.0001*
	II	40	5		21	
	III	8	5		6	
Histological grade	Grade I	3	0	.6185	1	.8171
	Grade II	54	8		23	
	Grade III	14	3		4	
CEBPD expression (CAF)	Low Exp. (<medium)	36	1	.0002*	5	<.0001*
	High Exp. (≧medium)	36	10		23	
PTX3 expression (CAF)	Low Exp. (<medium)	36	1	<.0001*	2	<.0001*
	High Exp. (≧medium)	36	10		26	

Abbreviations: CAF, cancer‐associated fibroblast; CEBPD, CCAAT/enhancer binding protein delta; Exp., expression; PTX3, pentraxin 3.

* Statistically significant.

**TABLE 2:** Correlation between CCAAT/enhancer binding protein delta (CEBPD) and pentraxin 3 (PTX3) expression and various clinicopathological factors in 72 triple‐negative breast cancer (TNBC) patients

			CEBPD expression (CAF)			PTX3 expression (CAF)		
Parameters	Category	No. of case	Low	High	*p*‐Value	Low	High	*p*‐Value
Age (years)	<60	57	31	26	.147	31	26	.147
	≧60	15	5	10		5	10	
Primary tumour (T)	T1	29	20	9	.006*	25	4	<.001*
	T2	34	15	19		11	23	
	T3–T4	9	1	8		0	9	
Nodal status (N)	N0	49	29	18	.006*	32	15	<.001*
	N1–N2	25	7	18		4	21	
Stage	I	24	17	7	.002*	22	2	<.001*
	II	40	19	21		14	26	
	III	8	0	8		0	8	
Histological grade	Grade I	3	1	10	.739	2	1	.203
	Grade II	54	27	71		29	25	
	Grade III	14	8	20		4	10	
PTX3 expression (CAF)	Low Exp. (<medium)	36	28	8	<.001*			
	High Exp. (≧medium)	36	8	28				

Abbreviations: CAF, cancer‐associated fibroblast; Exp., expression.

* Statistically significant.

**TABLE 3:** Multivariate survival analysis in 72 triple‐negative breast cancer (TNBC) patients

		DSS			MeFS		
Parameter	Category	H.R	95% CI	*p*‐Value	H.R	95% CI	*p*‐Value
Stage	I	1	–	.254	1	–	.230
	II–III	1.696	0.135–21.286		4.188	0.422–41.592	
	III	4.626	0.333–64.264		5.913	0.683–51.209	
CEBPD expression (CAF)	Low Exp. (<medium)	1	–	.140	1	–	.035*
	High Exp. (≧medium)	5.625	0.568–55.651		3.173	1.085–9.277	
PTX3 expression (CAF)	Low Exp. (<medium)	1	–	.267	1	–	.012*
	High Exp. (≧medium)	4.484	0.317–63.487		8.324	1.589–43.608	

Abbreviations: CAF, cancer‐associated fibroblast; CEBPD, CCAAT/enhancer binding protein delta; CI, confidence interval; DSS, disease‐specific survival; Exp., expression; MeFS, metastasis‐free survival; PTX3, pentraxin 3.

* Statistically significant.

**FIGURE 2:**
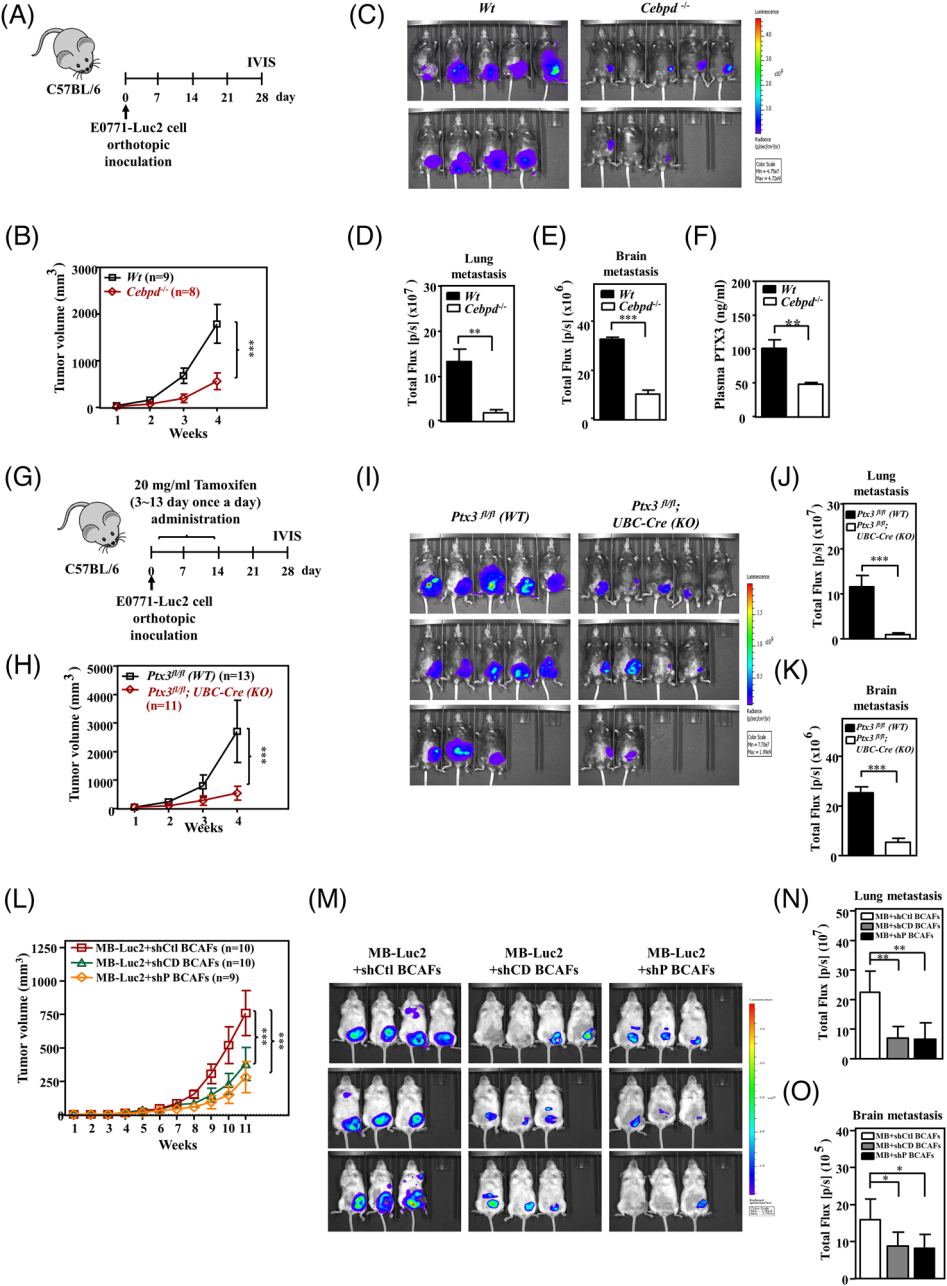
The growth and metastasis of triple‐negative breast cancer (TNBC) tumours are attenuated in *Cebpd*‐ and *Ptx3*‐deficient mice. (A) An experimental scheme for evaluating orthotopically allografted mouse TNBC luciferase‐expressing E0771 cells (E0771‐Luc2) in C57BL/6 (WT) and *Cebpd*‐deficient (*Cebpd^–/–^*) mice. (B) Growth of E0771‐Luc2 tumours in WT (*n* = 9) and *Cebpd^–/–^* (*n* = 8) mice was measured using external calipers. (C) Representative in vivo bioluminescence images and (D) metastasis quantification of E0771‐Luc2 tumours in the lung and (E) brain in experimental mice. (F) Plasma was collected from E0771‐Luc2‐bearing mice in the 4th week. Plasma pentraxin 3 (PTX3) was measured by ELISA. (G) An experimental scheme for evaluating orthotopically allografted E0771‐Luc2 tumours in tamoxifen‐induced conditional *Ptx3*‐knockout mice. (H) Growth of E0771‐Luc2 tumours in *Ptx3^fl/fl^* (WT) (*n* = 13) and *Ptx3^fl/fl^*; *UBC*‐Cre (KO) (*n* = 11) mice was measured. (I) Representative in vivo bioluminescence images and (J) metastasis quantification of E0771‐Luc2 tumours in the lung and (K) brain in experimental mice. (L) Tumour growth of MDA‐MB‐231‐Luc2 cancer cells (MB‐Luc2) co‐inoculated with breast cancer‐associated fibroblasts (BCAFs) bearing shVoid (shCtl‐BCAFs), shCEBPD (shCD‐BCAFs) or shPTX3 (shP‐BCAFs) vectors was measured in immunodeficient nonobese diabetic/severe combined immunodeficiency (NOD‐SCID) mice. (M) Representative in vivo bioluminescence images and (N) metastasis quantification of MB‐Luc2 tumours in the lung and (O) brain in experimental mice. All data are expressed as the mean ± SEM. Differences among the groups were analysed using unpaired two‐tailed *t*‐tests or one‐way ANOVA followed by Tukey's multiple comparison test. *p < .05, **p < .01, ***p < .001

To further assess the contribution of stromal CEBPD and PTX3 to the progression of TNBC, allografted mouse TNBC luciferase‐expressing E0771 cells (E0771‐Luc2) were developed for use in *Cebpd*‐deficient mice. The allograft E0771‐Luc2 tumours exhibited significantly attenuated tumour growth and metastasis in *Cebpd*‐deficient mice compared to wild type (WT) mice (Figure 2A–E). These results support our previous suggestion that stromal CEBPD plays a protumour role. Importantly, PTX3 levels in the blood of experimental mice bearing allograft E0771‐Luc2 tumours were correlated with the expression of CEBPD (Figure 2F) and were positively associated with growth and metastasis. These results further support the hypothesis that CEBPD is a PTX3 upstream regulator in the stroma.

To verify the protumour effect of stromal PTX3 on tumour growth and metastasis/invasion of TNBC, inducible conditional *Ptx3*‐deficient mice were generated (Figure S1A,B). In E0771‐Luc2 tumour‐bearing tamoxifen‐induced conditional *Ptx3‐*deficient mice, PTX3 expression was verified (Figure S1C) and attenuated PTX3 significantly reduced tumour growth and metastasis (Figure 2G **–**K).

CAFs constitute the majority of stromal cells and contribute to tumour growth and metastasis in breast carcinoma.^35^ We assessed the involvement in TNBC of CEBPD and PTX3 expression in breast cancer‐associated fibroblasts (BCAFs). For this reason, luciferase‐expressing MDA‐MB‐231 cells (MB‐Luc2) were co‐implanted with BCAFs carrying a void knockdown vector (shCtl‐BCAFs), a CEBPD knockdown vector (shCD‐BCAFs), or a PTX3 knockdown vector (shP‐BCAFs) into NOD‐SCID mice. Compared to MB‐Luc2/shCtl‐BCAFs grafts, the sizes of both MB‐Luc2/shCD‐BCAFs‐ and MB‐Luc2/shP‐BCAFs‐grafted tumours were significantly reduced and exhibited low metastatic activity (Figure 2L–O). These results support that stromal CEBPD and PTX3 play a protumour role in TNBC progression.

### 2.2 The BCAFs CEBPD/PTX3 axis mediates TGF‐β1‐induced stemness and metastasis of TNBC MDA‐MB‐231 cells

**FIGURE 3:**
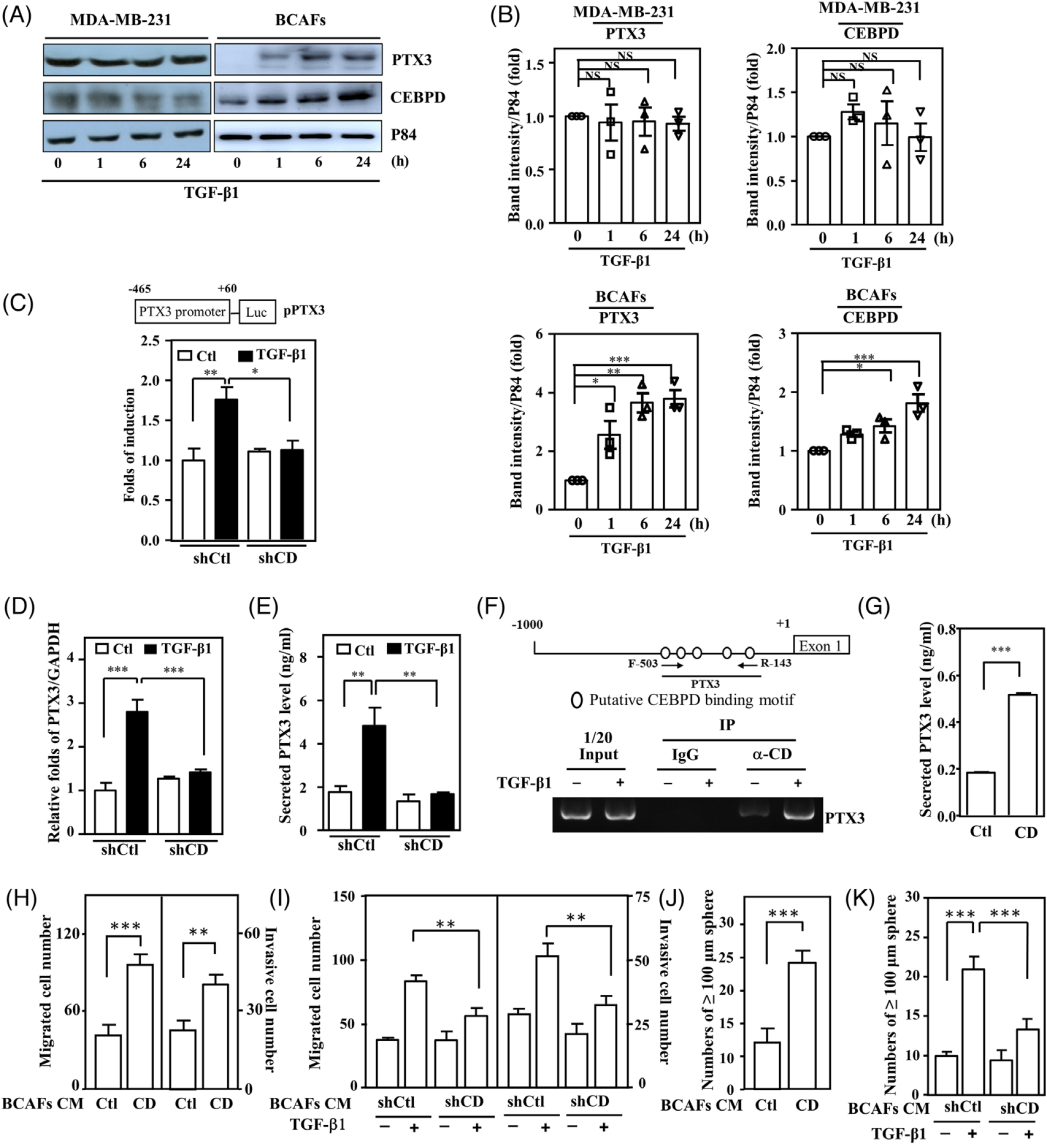
Pentraxin 3 (PTX3) transcription is responsive to TGF‐β1‐induced CCAAT/enhancer binding protein delta (CEBPD) in breast cancer‐associated fibroblasts (BCAFs), which promotes the migration, invasion and stemness of MDA‐MB‐231 cells. (A) Western blotting was performed to assess CEBPD and PTX3 expression in TGF‐β1‐treated MDA‐MB‐231 cells and BCAFs. p84 was used as an internal control. (B) Quantitative analysis of the levels of CEBPD and PTX3 in TGF‐β1‐treated MDA‐MB‐231 cells and BCAFs. (C) After pre‐incubation with shVoid (shCtl) or shCEBPD (shCD) lentivirus, a reporter assay was performed using lysates from TGF‐β1‐treated BCAFs transfectants. (D) PTX3 transcripts were examined with quantitative polymerase chain reaction (Q‐PCR) analysis. Total RNA was harvested from TGF‐β1‐treated BCAFs infected with shCtl or shCD lentiviruses. (E) The supernatant of TGF‐β1‐treated BCAFs infected with shCtl or shCD lentiviruses was harvested to examine conditioned medium PTX3 by ELISA. (F) Chromatin immunoprecipitated and PCR were performed using purified antibody‐immunoprecipitated fragmented gDNA from TGF‐β1‐treated BCAFs. (G) Conditioned media from BCAFs infected with lentiviruses bearing an empty vector (Ctl) or CEBPD cDNA (CD) were harvested to examine PTX3 by ELISA. (H) The migration and invasion of MDA‐MB‐231 cells were assessed using Transwell assays in which cells were cultured in conditioned medium from BCAFs infected with lentiviruses bearing an empty vector (Ctl) or CEBPD cDNA (CD). (I) Migration and invasion of MDA‐MB‐231 cells were assessed using a Transwell assay. The experimental cells were cultured in conditioned medium from BCAFs infected with shCtl and shCD lentiviruses and treated with or without TGF‐β1. (J) For the sphere formation assay, MDA‐MB‐231 cells were cultured in conditioned medium from BCAFs infected with lentiviruses bearing the expression vector Ctl or CD. (K) A sphere formation assay was conducted using MDA‐MB‐231 cells. The experimental cells were cultured in conditioned medium with or without TGF‐β1‐treated CAFs infected with lentiviruses bearing shCtl or shCD. All data are expressed as the mean ± SEM. Differences among groups were analysed using one‐way ANOVA followed by Tukey's multiple comparison test. *p < .05, **p < .01, ***p < .001, ns: no significance. Abbreviations: IP: Immunoprecipitation; RT‐PCR, reverse transcription polymerase chain reaction

A previous study suggested that CEBPD is responsive to TGF‐β1 in pancreatic stellate cells (myofibroblast‐like cells).^36^ Although CEBPD can upregulate PTX3 transcription in chemotherapy‐treated fibroblasts,^30^ the issue of whether TGF‐β1 can regulate CEBPD in BCAFs remains elusive. The involvement of TGF‐β1 and CEBPD in metastasis, promoting stemness and chemoresistance in cancer cells, has been previously suggested.^30^, ^37^ However, the issue of whether they can cross‐talk and be involved in the contribution of CAFs to breast cancer development remains elusive. We examined whether CEBPD and PTX3 were responsive to TGF‐β1 in BCAFs and found that CEBPD and PTX3 were responsive to TGF‐β1 in BCAFs but not in MDA‐MB‐231 cells (Figure 3A,B). Compared to various breast cancer cells, PTX3 expression and its response to TGF‐β1 were significantly higher in BCAFs (Figure S2). This result implies that PTX3 in breast cancers is primarily produced by BCAFs, so we focused on the regulatory mechanism of CEBPD and PTX3 in BCAFs.

The inactivation of CEBPD attenuated TGF‐β1‐induced *PTX3* promoter activity, transcription and secretion by BCAFs (Figure 3C–E). An in vivo DNA binding assay demonstrated that TGF‐β1 enhances CEBPD binding to the *PTX3* promoter in BCAFs (Figure 3F). Taken together, these results suggest that PTX3 expression is responsive to TGF‐β1‐enhanced CEBPD binding and activation in BCAFs. To further assess whether the BCAFs CEBPD/PTX3 axis contributes to TGF‐β1‐promoted TNBC malignancy, gain‐ and loss‐of‐function assays were performed. Conditioned media from BCAFs exogenously expressing CEBPD or TGF‐β1‐treated BCAFs could enhance the migration and invasion of MDA‐MB‐231 cells, but this phenomenon was attenuated by incubation with conditioned medium from TGF‐β1‐treated shCEBPD‐BCAFs (Figure 3G–I). We also assessed the relationship of TGF‐β1‐induced CEBPD activation in BCAFs and its contribution to cancer stemness. The results of the in vitro sphere formation assay showed that conditioned medium from BCAFs ectopically expressing CEBPD enhanced the stemness of MDA‐MB‐231 cells (Figure 3J). Moreover, the in vitro stemness activity of MDA‐MB‐231 cells was enhanced upon incubation with conditioned medium from TGF‐β1‐treated BCAFs but was attenuated in medium from TGF‐β1‐treated shCEBPD‐BCAFs (Figure 3K). These results suggest that PTX3 is responsive to TGF‐β1‐induced CEBPD in BCAFs and contributes to promoting the migration, invasion and stemness of TNBC MDA‐MB‐231 cells.

### 2.3 PTX3‐responsive genes are known to be involved in stemness, bone invasion and matrix turnover

PTX3 is responsive to TGF‐β1 via CEBPD‐mediated direct regulation in BCAFs. To understand the effectors responding to PTX3 in cancer progression, a whole‐genome transcriptomic analysis was performed with transcription products harvested from PTX3‐treated MDA‐MB‐231 cells. Using clusterProfiler, we identified 3952 overlapping genes in the gene profiles at 24 h and 1 week (Figure S3A) to characterise the PTX3‐participating functional roles and gene clusters. In this gene set, we found that PTX3‐responsive genes were primarily associated with the regulation of cell motility, morphogenesis of the epithelium, and osteoblast differentiation and development (Figure S3B,C). Among them, in PTX3‐treated MDA‐MB‐231 cells, we confirmed transcriptional activation of stemness genes (e.g. *Twist1/2*), developmental process regulators (e.g. *Nanog*), bone invasion (e.g. *RANKL*) and matrix modifiers (e.g. *MMP2*) (Figure S3D).

### 2.4 PTX3 directly interacts with CD44 to induce the migration, invasion and stemness of MDA‐MB‐231 cells

**FIGURE 4:**
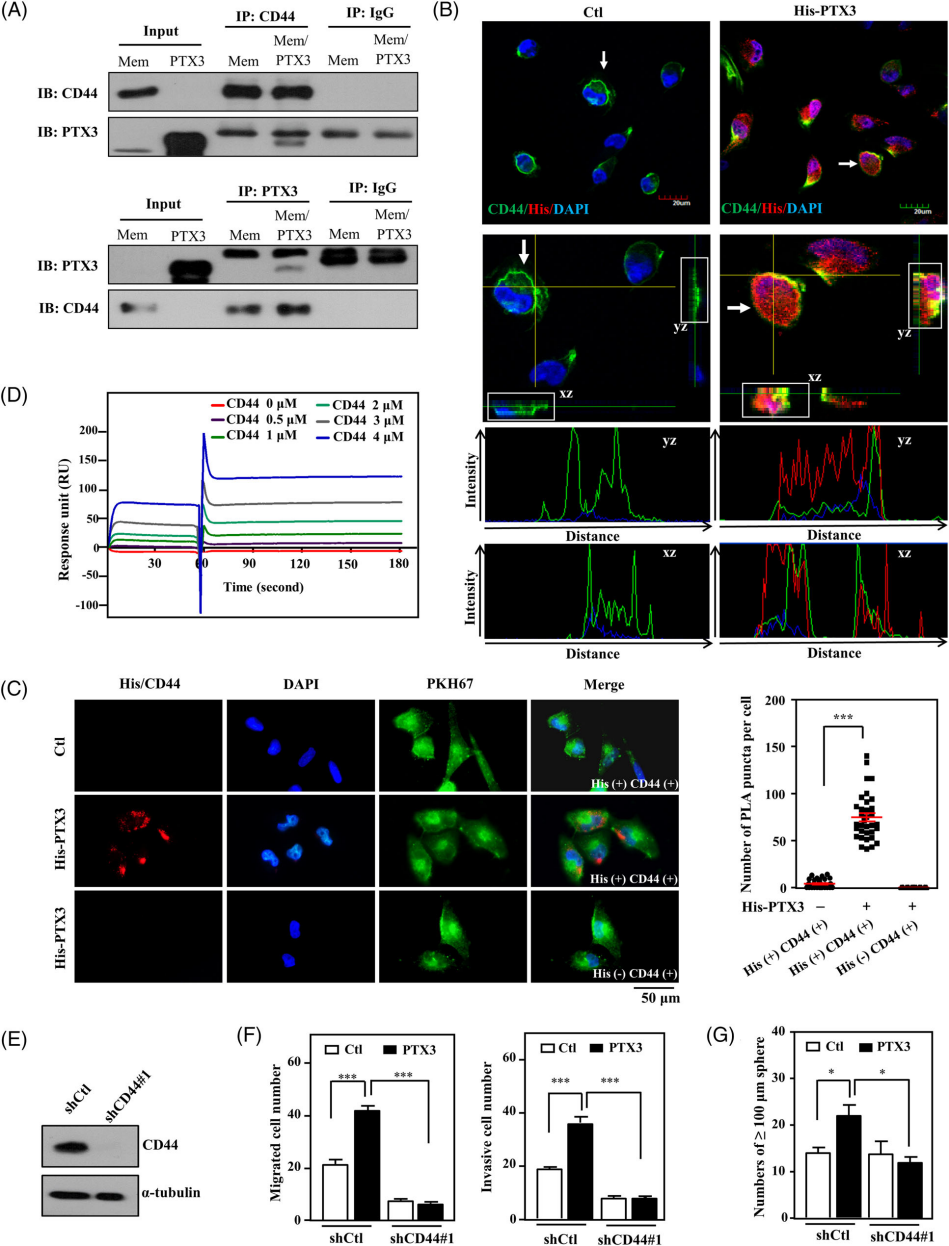
Pentraxin 3 (PTX3) interacts with CD44 and induces the migration, invasion and stemness of MDA‐MB‐231 cells. (A) Harvested MDA‐MB‐231 cell membrane proteins were incubated with or without recombinant PTX3 protein, and then co‐immunoprecipitation was performed with the membrane proteins and PTX3 antibody or CD44 antibody. Immunoglobulin G (IgG) was used as a negative control. (B) PTX3‐treated MDA‐MB‐231 cells were immobilised and detected by immunofluorescence using confocal microscopy after staining with the indicated antibodies. Representative immunostaining of MDA‐MB‐231 cells with merged two‐dimensional images. (C) The interaction between His‐PTX3 and CD44 was determined by in situ proximity ligation assay (PLA) using anti‐His and anti‐CD44 antibodies (His+, CD44+). The red spots represent interacting complexes of His‐PTX3 and CD44. Cells stained with anti‐CD44 antibody only (His‐, CD44+) were used as a negative control. The nuclei were stained with DAPI (blue). The cell membrane was stained with PKH67. Protein interactions were quantified by counting the number of puncta per cell. (D) The binding affinity of PTX3 to CD44 was assayed using PTX3‐immobilised BIAcore sensor chips passed through CD44. The interaction was monitored by surface plasmon resonance. (E) MDA‐MB‐231 cells were infected with lentiviral control (shCtl) or shCD44#1. Lysates from experimental cells were harvested for Western blotting. (F) Migration and invasion of lentiviral shCtl‐ or shCD44#1‐infected MDA‐MB‐231 cells with or without PTX3 were assessed using a Transwell assay. (G) The number of shCtl or shCD44#1 tumour sphere of MDA‐MB‐231 cells was counted after 7 days of incubation with or without PTX3 treatment. All data are expressed as the mean ± SEM. Differences among groups were analysed using one‐way ANOVA followed by Tukey's multiple comparison test. *p < .05, ***p < .001

Accumulating evidence in conjunction with our current results indicates that PTX3 is associated with or contributes to cancer malignancies. Methylation of the *PTX3* promoter and loss of CEBPD expression typically result in attenuated PTX3 expression in cancer cells.^38^, ^39^ However, the activation of stromal PTX3, including fibroblast PTX3, can still support cancer progression. In addition, the receptor directly responding to PTX3 binding and mediating PTX3‐induced protumour effects in cancer cells remains unknown. Using recombinant His/PTX3 from Expi293F human cells as bait, we explored PTX3 receptors by pull‐down assays and liquid chromatography‐tandem mass spectrometry (LC–MS/MS). Among the potential PTX3 receptors, CD44 was the focus because of its confirmed involvement in tumour recurrence, mortality, metastasis and invasion in malignant cancers.^18^, ^40^, ^41^, ^42^ We tested whether CD44 could function as a receptor for direct PTX3 binding and contribute to PTX3‐induced metastasis and stemness in MDA‐MB‐231 cells. A co‐immunoprecipitation assay demonstrated that recombinant PTX3 protein directly interacts with CD44 on the MDA‐MB‐231 cell membrane (Figure 4A). We next performed an immunofluorescence assay and showed that recombinant PTX3 proteins were co‐localised with CD44 on the MDA‐MB‐231 cell membrane (Figure 4B). Moreover, an in situ proximity ligation assay (PLA) was performed to verify whether recombinant PTX3 binds to CD44 and associates with CD44 activity. The fluorescent signals could be observed after binding to the amplified DNA following the interaction of the active form of CD44^43^ and recombinant PTX3 on the cell membrane of MDA‐MB‐231 cells (Figure 4C). These results suggested that PTX3 can directly interact with activated CD44 on the cell membrane. We validated PTX3 directly interacting with CD44 by using surface plasmon resonance technology. The binding kinetics results indicated that the N‐terminus of CD44 directly interacts with full‐length PTX3 immobilised on a chip. The association rate constant, *K*_on_, and the dissociation rate constant, *K*_off_, of the N‐terminal CD44 and full‐length PTX3 binding were 1.30 ± 0.32 × 10^2^ M^–1^ s^–1^ and 2.43 ± 2.35 × 10^–6^ s^–1^, respectively (mean ± standard deviation). Meanwhile, the equilibrium dissociation constant, *K*_D_, was calculated as 0.021 ± 0.012 μM (mean ± standard deviation), indicating a high‐affinity binding interaction between PTX3 and CD44 (Figure 4D). The above results all indicate that, as a ligand, PTX3 can directly and specifically interact with CD44.

We next examined whether CD44 mediates PTX3‐induced migration, invasion and stemness of MDA‐MB‐231 cells. A short hairpin RNA (shRNA)‐based approach was utilised to generate CD44 knockdown MDA‐MB‐231 cells (shCD44/MB231). After confirming reduced CD44 expression in stable shCD44/MB231 cells (Figures 4E and S4A), we found that depletion of CD44 attenuates PTX3‐induced migration, invasion and stemness of MDA‐MB‐231 cells (Figures 4F–G and S4B,C). In addition to revealing that CD44 is a novel PTX3 receptor, these findings indicate that CD44 not only directly interacts with PTX3 but also makes an important impact on PTX3‐induced protumour activities in MDA‐MB‐231 cells.

### 2.5 As a novel PTX3 receptor, CD44 specifically mediates PTX3‐induced ERK1/2, AKT and NF‐κB signalling pathways in MDA‐MB‐231 cells

**FIGURE 5:**
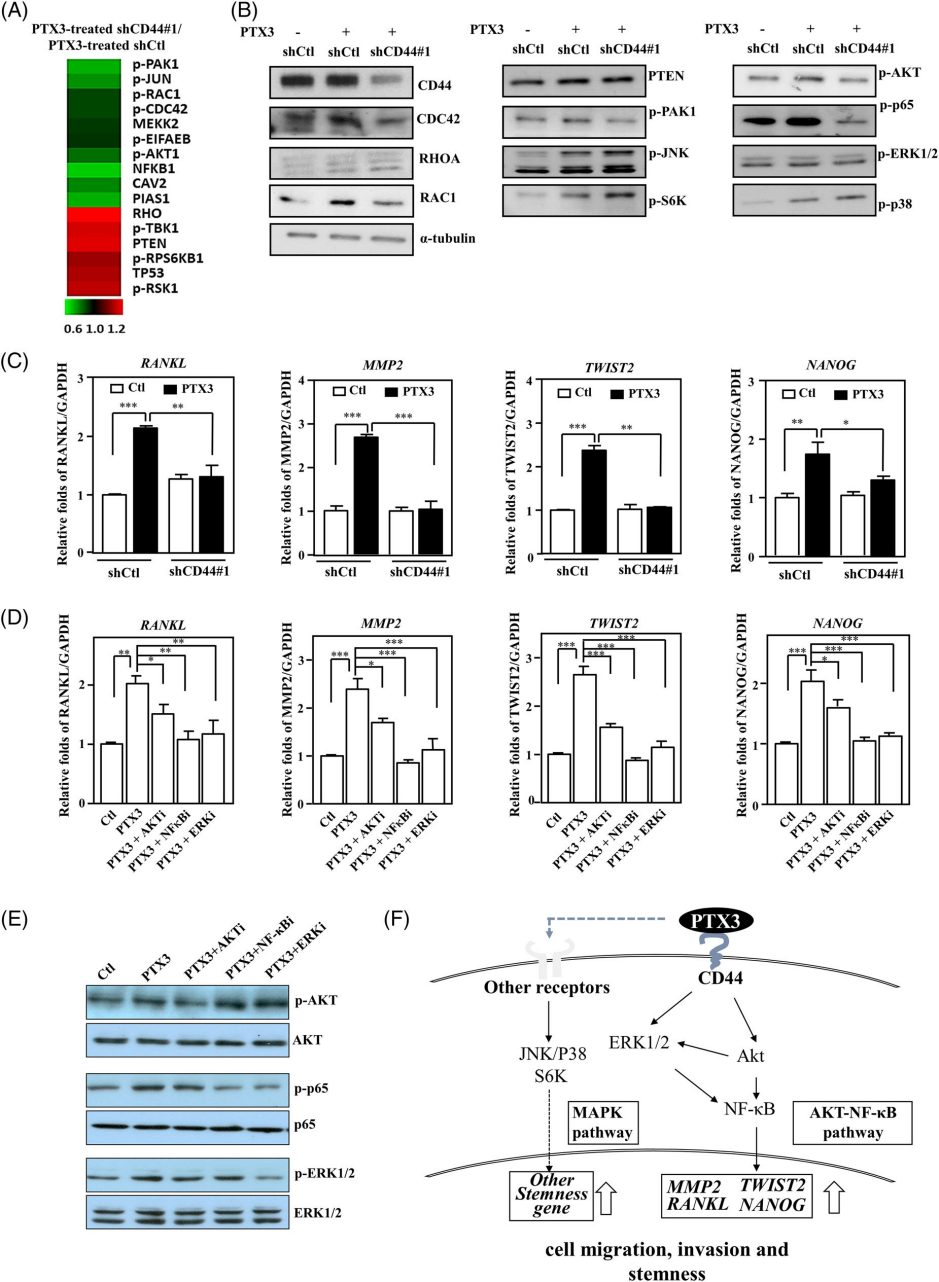
Pentraxin 3 (PTX3) interacts with CD44 and regulates metastatic and stemness gene expression through the ERK1/2, AKT and NF‐κB pathways in MDA‐MB‐231 cells. (A) MDA‐MB‐231 cells were pre‐infected with control (shCtl) or shCD44#1 lentivirus and then treated with PTX3 without serum in the medium. Lysates from the experimental cells were harvested for microwestern array analysis. The quantitative results of the microwestern array analysis were obtained and are presented as the ratio of protein levels in PTX3‐treated shCD44 MDA‐MB‐231 cells/PTX3‐treated shCtl MDA‐MB‐231 cells. Expression of the indicated proteins was normalised to the average of α‐tubulin first and then individually themselves, and the increased or decreased protein levels are represented as red or green, respectively. (B) MDA‐MB‐231 cells were pre‐infected with lentivirus bearing shCtl or shCD44#1 expression vector and were then treated with PTX3 for 30 min. Lysates from the experimental cells were harvested for Western blot analysis, and specific antibodies were applied, as indicated. Relative protein expression was normalised to α‐tubulin. (C) MDA‐MB‐231 cells were pre‐infected with lentivirus bearing shCtl or shCD44#1 vector and were then treated with PTX3 for 24 h. (D) MDA‐MB‐231 cells were pre‐treated with or without wortmannin (100 nM), BAY 11‐7085 (2 μM) or PD98059 (10 μM) for 1 h and were then treated in the presence or absence of PTX3 for 24 h. mRNA levels of *RANKL*, *MMP2*, *TWIST2* and *NANOG* were measured by real‐time reverse transcription polymerase chain reaction (RT‐PCR). *GAPDH* was used as an internal control. (E) The indicated signalling inhibitors were pre‐treated for 1 h and then treated in the presence or absence of PTX3 for 24 h. Total cell lysates were prepared and immunoblotted for p‐AKT, p‐p65 and p‐ERK1/2 and were normalised to the respective non‐phosphorylated protein levels. (F) A highly simplified diagram of the PTX3/CD44 signalling pathway. All data are expressed as the mean ± SEM. Differences among groups were analysed using one‐way ANOVA followed by Tukey's multiple comparison test. *p < .05, **p < .01, ***p < .001

Next, we explored PTX3/CD44‐induced signalling pathways in MDA‐MB‐231 cells using microwestern arrays. We treated shCD44/MB231 cells with PTX3 to verify the involvement of CD44 in PTX3‐induced signalling pathways. Compared to PTX3‐treated control knockdown MDA‐MB‐231 cells (shCtl/MB231), the signals of pPAK1, pJUN, pRAC1, pCDC42, MEKK2, pEIFAEB, pAKT1, NFKB1, CAV2 and PIAS1 were attenuated, but the signals of RHO, pTBK1, PTEN, pRPS6KB1, TP53 and pRSK1 were reversed in PTX3‐treated shCD44/MB231 cells (Figure 5A). We further verified the activation of p65, AKT, ERK1/2, p38 and c‐Jun N‐terminal kinase (JNK) in MDA‐MB‐231 cells via Western blotting. Compared to PTX3‐treated shCtl/MB231 cells, phosphorylation of ERK1/2, p65 and AKT, but not JNK or p38, was significantly decreased in PTX3‐treated shCD44/MB231 cells (Figures 5B and S5A). These results indicate that CD44 specifically mediates PTX3‐induced activation of ERK1/2, PI3K/AKT and NF‐κB signalling but not JNK, p38 or p70S6K signalling in MDA‐MB‐231 cells. Moreover, CD44 participates in PTX3‐induced *TWIST2*, *NANOG, RANKL* and *MMP2* transcription in MDA‐MB‐231 cells (Figures 5C and S4D).

We then assessed whether the ERK1/2, PI3K/AKT and NF‐κB pathways contributed to PTX3‐induced *RANLK*, *TWIST2*, *MMP2* and *NANOG* transcription. Specific pharmacologic inhibitors of PI3K/AKT (wortmannin), NF‐κB (BAY 11‐7085) and MEK1/ERK1/2 (PD98059) were applied to address this issue. Inhibition of the PI3K/AKT, NF‐κB and MEK1/ERK1/2 pathways in PTX3‐treated MDA‐MB‐231 cells significantly hampered *RANLK*, *TWIST2*, *MMP2* and *NANOG* transcription (Figure 5D). We also assessed the hierarchy of AKT, NF‐κB and ERK1/2 using specific signalling cascade inhibitors in PTX3‐treated MDA‐MB‐231 cells (Figures 5E and S5B). These results showed that AKT activation contributes to ERK1/2 activation and consequently affects NF‐κB activation. To clarify the regulation that occurs in response to PTX3 and CD44 in MDA‐MB‐231 cells, a schematic illustration was created to summarise these results (Figure 5F).

### 2.6 Targeting the C‐terminus of PTX3 prevents stemness, migration and invasion of MDA‐MB‐231 cells

PTX3 enhanced the migration and invasion of MDA‐MB‐231 cells (Figure S6A–C) and promoted their sphere‐forming ability (Figure S6D) in vitro. A single N‐glycosylation site has been identified in the C‐terminal domain of *PTX3* at *Asn220*.^20^ We found that a recombinant PTX3 Asn220 mutant (PTX3NA) had no effect on the sphere formation, migration or invasion of MDA‐MB‐231 cells. Importantly, PTX3NA dramatically inhibited all of the above PTX3‐induced phenomena (Figure S6A–D). This implies that PTX3 glycosylation is important for its protumour roles and PTX3NA could be an antagonist that competes with PTX3 binding to the PTX3 receptor to inhibit PTX3‐induced protumour functions. In addition to PTX3NA, recombinant truncated mutants of PTX3 that included either the N‐terminus of PTX3 (PTX3/N, amino acids 19–182) or the C‐terminus of PTX3 (PTX3/C, amino acids 180–381) were produced (Figure S7A). The results of in vitro protein binding assays showed that CD44 could bind to PTX3, PTX3NA and PTX3/C but not to PTX3/N (Figure S7B). These results indicate that the glycosylation of PTX3 Asn220 has no effect on the binding of PTX3 and CD44, but the C‐terminus of PTX3 is a critical and important region for the direct interaction between PTX3 and CD44. Next, we examined whether the functional domain of PTX3 contributes to the metastasis and invasion of MDA‐MB‐231 cells. We found that PTX3/C mimics PTX3 to induce the metastasis and invasion of MDA‐MB‐231 cells (Figure S7C). Taken together, these results imply the involvement of the C‐terminus of PTX3 in the CD44 interaction and in the PTX3‐induced migration and invasion of MDA‐MB‐231 cells.

To further dissect the specific binding region of the PTX3 C‐terminus that interacts with CD44, computer modelling was performed using the structures of the highly conserved pentraxin domains of the known PTX3 family members serum amyloid P component (SAP) and C‐reactive protein (CRP) and the contact surface region between the PTX3 C‐terminus and CD44 for prediction. Peptides RI37 (PTX3 amino acids 200–236)^30^ and GI40 (PTX3 amino acids 320–359) were generated and assessed to verify the interaction interface between PTX3 and CD44 and its effect on the metastasis/invasion of cancer cells (Figure S7D). In a competition assay, the binding of PTX3 and CD44 was attenuated by competition with RI37 and GI40 peptides (Figure S7E). This further confirmed that the C‐terminus of PTX3 is responsible for binding to CD44. We next assessed whether these peptides attenuate signal transduction and the consequent protumour effects from the interaction of PTX3 and CD44, including invasion and stemness. The results showed that both RI37 and GI40 suppressed PTX3‐induced AKT, NF‐κB and ERK1/2 activity (Figure S7F) and transcription of *RANLK*, *MMP2*, *NANOG* and *TWIST2* in MDA‐MB‐231 cells (Figure S7G). Moreover, the RI37 and GI40 peptides suppressed the PTX3‐induced sphere‐forming ability and the in vitro migration and invasion of MDA‐MB‐231 cells (Figure S7H,I). Furthermore, we tested the antitumour effect of the RI37 and GI40 peptides in vivo using a xenograft model. The results showed that the tumour growth and metastasis of mCherry fluorescent‐expressing MDA‐MB‐231 cells and 4T1 cells in mice were suppressed in the group of experimental mice intraperitoneally injected with RI37 and GI40 peptides (Figure S7J,K). This finding indicates that RI37 and GI40 induce antitumour activity to suppress PTX3‐induced cancer growth and metastasis.

### 2.7 Shortened PTX3 peptides exert an antitumour effect by disrupting the PTX3/CD44 interaction

**FIGURE 6:**
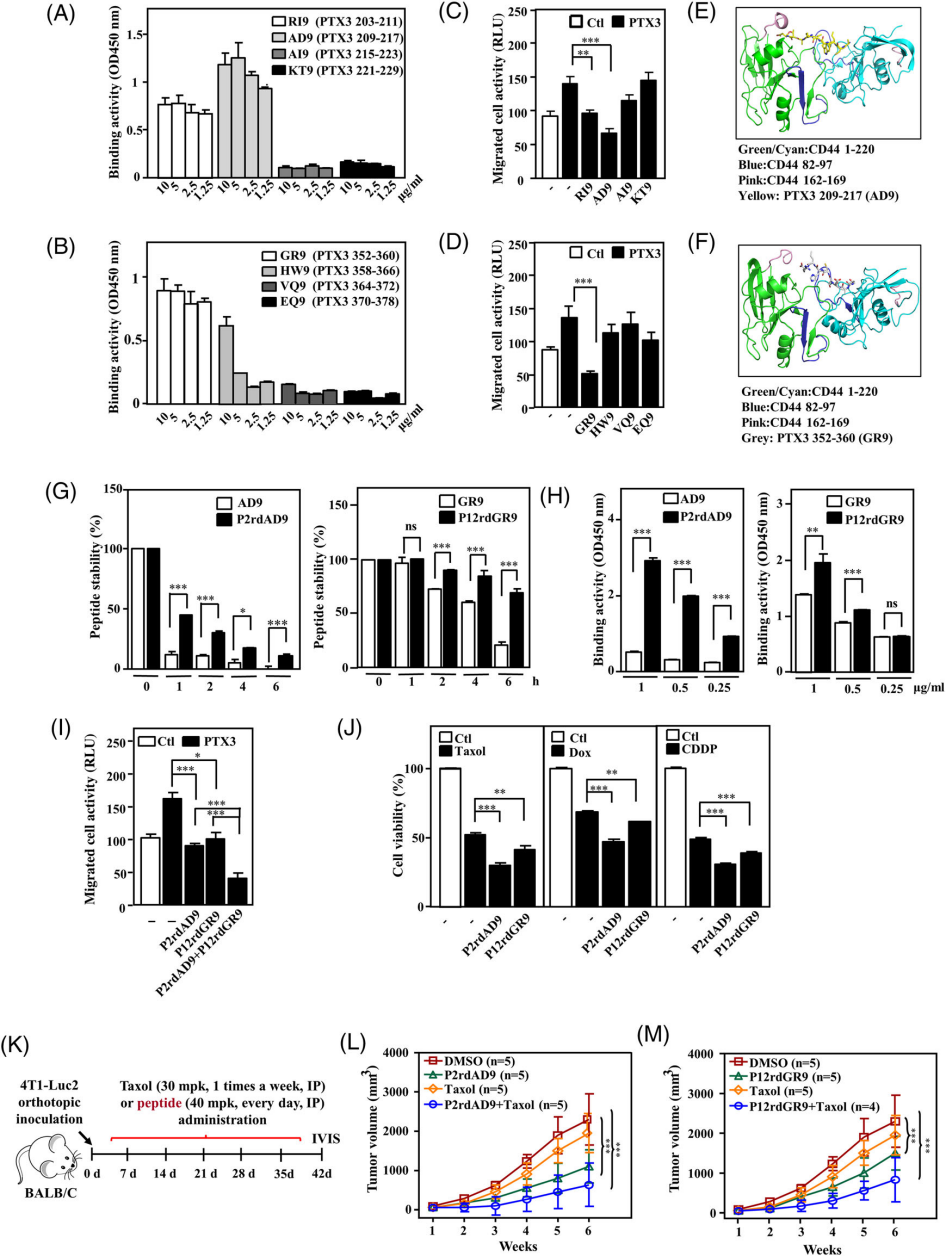
A combination of pentraxin 3 (PTX3) peptide and paclitaxel enhances the suppressive effect on tumour growth and metastasis in a triple‐negative breast cancer (TNBC) mouse model. (A and B) The activity of various biotinylated truncated PTX3 peptides to bind immobilised CD44 was determined using ELISA. (C and D) The migration of various biotinylated truncated PTX3 peptide‐treated luciferase‐expressing 4T1 cells (4T1‐Luc2) with or without PTX3 treatment was assessed using the Transwell assay. 4T1‐Luc2 cells were assessed for migration ability by measuring luciferase activity. Protein remodelling predicted the interaction of (E) AD9 and (F) GR9 with the CD44 dimer. (G) The stability of various biotinylated and PEGylated retro‐inverso narrowed down PTX3 peptides AD9 (P2rdAD9) or GR9 (P12rdGR9) was assessed by incubation with serum, and their stability was analysed at the indicated times by ELISA. Peptide levels were calculated relative to the quantities determined at time point zero. (H) The CD44 binding activity of P2rdAD9 or P12rdGR9 was assessed by incubation with CD44, and their interaction was analysed by ELISA. (I) The effect of P2rdAD9 or P12rdGR9 on the migration of PTX3‐treated 4T1‐Luc2 cells was assessed using a Transwell assay, as indicated. (J) The effect of P2rdAD9 or P12rdGR9 on the cytotoxicity of anticancer drug‐treated 4T1 cells was assessed using 3‐(4,5‐Dimethylthiazol‐2‐yl)‐2,5‐diphenyltetrazolium bromide (MTT) assay. Paclitaxel (Taxol), doxorubicin (Dox) and cisplatin (CDDP). (K) An experimental scheme for evaluating orthotopically allografted 4T1‐Luc2 tumours in BALB/c mice. Once tumours reached an average volume of 50 mm^3^, the mice were administered P2rdAD9 or P12rdGR9, with or without Taxol. (L and M) The effect of P2rdAD9, P12rdGR9 or Taxol on the growth of 4T1‐Luc2 tumours in BALB/c mice was measured using external calipers. All data are expressed as the mean ± SEM. Differences among groups were analysed using one‐way ANOVA followed by Tukey's multiple comparison test or unpaired two‐tailed *t*‐test. *p < .05, **p < .01, ***p < .001, ns: no significance

To further identify the critical region for disrupting the interaction between PTX3 and CD44, sequentially shorter PTX3 peptides were designed to verify their inhibitory effect and antitumour activity. Among them, AD9 (PTX3 amino acids 209–217) and GR9 (PTX3 amino acids 352–360) displayed significantly high binding activity with CD44 (Figure 6A,B) and efficiently inhibited PTX3‐induced migration of mouse TNBC luciferase‐expressing 4T1 cells (4T1‐Luc2) (Figure 6C,D). After confirming the critical functional sequences of PTX3, protein remodelling was used to predict the PTX3‐binding region on CD44. The prediction results indicated that AD9 and GR9 interact with CD44 amino acids 82–97 and 162–169, respectively (Figure 6E,F). Next, stable PEGylated retro‐inverso peptides AD9 (P2rdAD9) and GR9 (P12rdGR9) were produced, and their antitumour effect was assessed. P2rdAD9 and P12rdGR9 both exhibited better stability and increased CD44 binding activity (Figure 6G,H). Similar to their parental peptides, P2rdAD9 and P12rdGR9 inhibited PTX3‐induced 4T1‐Luc2 cell migration in vitro, and co‐treatment with P2rdAD9 and P12rdGR9 resulted in a better inhibitory effect (Figure 6I) and enhanced first‐line TNBC anticancer drug‐induced apoptosis (Figure 6J). In an allograft 4T1‐Luc2 tumour model, P2rdAD9 or P12rdGR9 with paclitaxel co‐treatment displayed a stronger inhibitory effect against tumour growth and metastasis/invasion than paclitaxel. Moreover, the effects of P2rdAD9 or P12rdGR9 combined with paclitaxel were better than those of the other treatment groups. Compared to P12rdGR9, P2rdAD9 exhibited better translational application potential (Figures 6K–M and S8A–D). In conclusion, the modified PTX3 peptide P2rdAD9 effectively inhibited tumour growth and metastasis and increased the efficacy of chemotherapeutic drugs.

### 2.8 PTX3 antibodies inhibit PTX3‐induced signalling and tumour progression in TNBC

**FIGURE 7:**
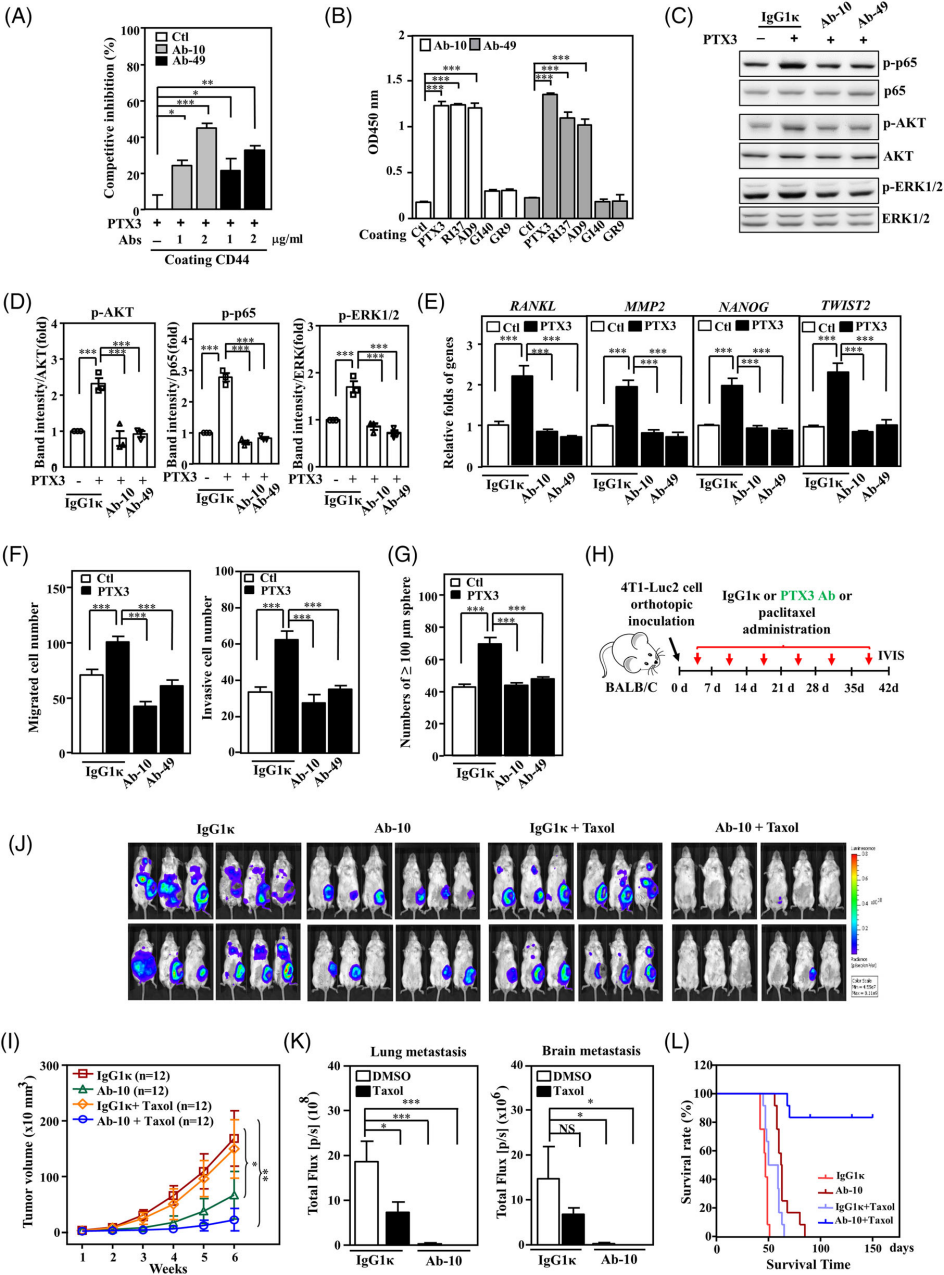
The effects of pentraxin 3 (PTX3) antibodies on triple‐negative breast cancer (TNBC) cells. (A) PTX3 antibodies (Ab‐10 or Ab‐49) inhibit the interaction between PTX3 and CD44. The abilities of PTX3‐ and PTX3 pre‐incubated with Ab‐10 or Ab‐49 to bind immobilised CD44 were assessed using a competitive binding assay. (B) Epitope identification by ELISA using the indicated PTX3 antibodies and immobilised PTX3 or various shortened PTX3 peptides; the PTX3 amino acids 200–236 (RI37), 320–359 (GI40), 209–217 (AD9) and 352–360 (GR9) were examined for recognition by Ab‐10 or Ab‐49. (C) The suppressive effect of PTX3 antibodies on PTX3‐activated signalling pathways in MDA‐MB‐231 cells. MDA‐MB‐231 cell lysates were incubated with isotype antibody (IgG1κ), Ab‐10 or Ab‐49 and harvested for immunoblotting with the indicated antibodies. (D) Quantitative analysis of the protein activity of p‐AKT, p‐p65 and p‐ERK1/2 in MDA‐MB‐231 cells. (E) The suppressive effect of PTX3 antibodies on PTX3‐activated gene transcription in MDA‐MB‐231 cells. The total RNA of MDA‐MB‐231 cells that were incubated with IgG1κ, Ab‐10 or Ab‐49 was harvested for real‐time reverse transcription polymerase chain reaction (RT‐PCR). (F) The migration and invasion of IgG1κ‐, Ab‐10‐ and Ab‐49‐treated MDA‐MB‐231 cells with or without PTX3 treatment were assessed by Transwell assay. (G) An in vitro sphere formation assay was performed with IgG1κ‐, Ab‐10‐ and Ab‐49‐treated MDA‐MB‐231 cells with or without PTX3 treatment. (H) IgG1κ, PTX3 antibody (Ab‐10) and paclitaxel (Taxol) in the indicated groups of orthotopically allografted 4T1‐Luc2‐bearing mice by intraperitoneal injection. (I) The effects of IgG1κ, Ab‐10 and Taxol on the growth of 4T1‐Luc2 tumours in BALB/c mice were measured using external calipers. (J) Representative in vivo bioluminescence images, (K) metastasis quantification of 4T1‐Luc2 tumours in lung and brain and (L) Kaplan–Meier survival curves after administering IgG1κ, PTX3 antibody (Ab‐10) or paclitaxel (Taxol) in the indicated groups of 4T1‐Luc2‐bearing mice. All data are expressed as the mean ± SEM. Differences among groups were analysed using one‐way ANOVA followed by Tukey's multiple comparison test. *p < .05, **p < .01, ***p < .001, ns: no significance

Monoclonal antibodies that recognise the C‐terminus of PTX3 and disrupt the interaction between PTX3 and CD44 were developed to validate the PTX3/CD44 axis‐induced protumour effects on cancer development, metastasis/invasion and stemness. The antibodies were screened (Figure S9), verified to inhibit PTX3/CD44 binding activity (Figure 7A), and the two most effective antibodies, Ab‐10 and Ab‐49, were selected for further experiments. Interestingly, Ab‐10 and Ab‐49 recognised PTX3, RI37 and AD9 but not GI40 or GR9 (Figure 7B). To further assess whether neutralisation of fibroblast PTX3 attenuates TGF‐β1‐promoted MDA‐MB‐231 cell malignancy, IgG1κ‐treated MDA‐MB‐231 cells with or without conditioned medium from TGF‐β1‐treated BCAFs enhanced the migration/invasion and stemness of MDA‐MB‐231 cells, but this phenomenon was attenuated by incubation of MDA‐MB‐231 cells with Ab‐10 or Ab‐49 (Figure S10).

Similar to the effect of RI37 and P2rdAD9, in vitro assays showed that Ab‐10 and Ab‐49 also inhibited PTX3‐induced signalling pathways, the transcription of known downstream genes, and the cancer features of migration/invasion and stemness (Figure 7C–G). Importantly, Ab‐10 significantly blocked tumour growth and metastasis/invasion of allograft 4T1‐Luc2 tumours and xenograft MB‐Luc2 tumours and improved the survival rate (Figures 7H–L and S11A–D). These results suggest that Ab‐10 harbours strong antitumour activity to suppress the growth and metastasis of TNBC.

## 3 DISCUSSION

PTX3 is classified as an evolutionarily conserved pentraxin family member that participates in the regulation of innate immunity. During tumourigenesis, elevated PTX3 levels have been detected in various cancers, including liposarcoma, glioma, ovarian cancer, lung cancer, pancreatic carcinoma, prostate carcinoma and hepatocellular carcinoma, and are correlated with the grade of malignancy and a poor prognosis.^32^, ^33^, ^34^, ^44^, ^45^, ^46^, ^47^ Although these data together imply the pro‐tumourigenic role of PTX3 in tumour progression, as we suggested, these findings are in sharp contrast to other reports in which PTX3 was found to exert a tumour‐suppressive role. Briefly, the N‐terminus of PTX3 was suggested to inhibit angiogenesis and tumour growth in fibroblast growth factor (FGF)‐dependent murine prostate cancer and melanoma by binding to fibroblast growth factors, including FGF2 and FGF8b.^28^, ^29^ It was further demonstrated that FGF2‐dependent angiogenesis, tumour growth and metastasis were enhanced in *Ptx3*‐deficient mice. However, these observations were reversed in allograft FGF2‐dependent tumours in endothelial‐specific Tie2 promoter‐driven *hPTX3* transgenic mice.^48^ In fact, the results specifically indicate that PTX3 plays a role in suppressing the tumour initiation of FGF‐dependent tumours but not PTX3‐elevated cancers.

In addition to cancer cell complexity, a dynamic change in the TME can also develop after tumourigenesis. Recently, immunoediting, a dynamic process that consists of immunosurveillance and tumour progression, has been suggested to occur during tumourigenesis. Communication between cancer cells and stromal cells, including immune cells, remains largely uninvestigated. Inhibition of fibroblast growth factor receptors (FGFR) signalling leads to decreased growth of TNBC cells.^49^ Clinically applied FGFR inhibitors showed only a 4% benefit in TNBC patients.^50^ Moreover, in various cells, including cancer‐associated stromal cells, the transcription factor CEBPD is responsive to external stimuli, such as inflammatory factors, stressors and chemotherapeutic drugs.^25^, ^26^, ^30^, ^51^ In addition to PTX3, TSG‐6 (also called TNFAIP6) is a CEBPD‐responsive gene^52^ that is suggested to reverse the inhibitory effects exerted by the N‐terminus of PTX3 on FGF2‐mediated angiogenesis by competing with the FGF2/PTX3 interaction.^53^ These findings imply that CEBPD activation in the TME may allow escape of the PTX3‐inhibited FGF2 signalling pathway by inducing TSG‐6, which competes with the FGF2/PTX3 interaction. In other words, the abovementioned tumour‐suppressive role of the N‐terminus of PTX3 may rely on the existence of TSG‐6, especially in a CEBPD‐activated TME. However, this speculation requires further verification.

Our current results suggest that the C‐terminus of PTX3 directly interacts with the N‐terminus of CD44, consequently activating metastasis/invasion and stemness through the involved genes by activating the AKT, ERK1/2 and NF‐κB pathways in breast cancer cells. However, knockdown of CD44 enhanced both JNK and p38 activity, implying that blockade of the PTX3/CD44 interaction positively compensates for JNK and p38 activity. Although our current results suggest that CD44 is a novel PTX3 receptor and that the PTX3/CD44 complex does indeed play a vital role in the promotion of metastasis/invasion and stemness of cancer cells, we cannot rule out other potent PTX3 receptors and their involvement in tumourigenesis. Here, we demonstrated that PTX3 is a novel CD44 ligand. Therefore, understanding how PTX3 competes with other CD44 ligands, such as HA and TSG‐6, for CD44 binding is an interesting issue. Regarding previous studies, TSG‐6 and CD44 show a topologically conserved region, link modules, in their N‐terminus.^54^, ^55^ HA can interact with link modules on TSG‐6, but such interaction has no effect on the PTX3 interaction with TSG‐6.^56^ Therefore, PTX3 may not disturb the interaction between HA and CD44, at least in part, through link modules. In addition, TSG‐6 has been suggested to interact with PTX3.^53^ In addition to PTX3, TSG‐6 is a secretory protein.^57^ This result suggests that the link modules of TSG‐6 have the potential to compete with CD44 for PTX3 interaction. However, CD44 shows a higher affinity (KD = 0.021 μM) than TSG‐6 (KD = 0.314–0.648 μM) for PTX3 binding.^56^ Combined with our current study, these results suggest that TSG‐6 is a lower affinity competitor for PTX3 binding to CD44. In addition, no evidence shows that HA can directly interact with PTX3. In the current study, AD9 (PTX3 amino acids 209–217) and GR9 (PTX3 amino acids 352–360) interact with CD44 amino acids 82–97 (Figure 6E,F), which overlaps with the CD44 amino acids 38–105 for interacting with HA. This implies that short peptide inhibitors may affect the interaction between HA and CD44. However, the above observations need further investigation to address. In a previous study, PTX3 was found to directly interact with FcγR on the surface of leukocytes during phagocytosis.^58^ Activation of the FcγR pathway in myeloid cells was recently suggested to promote squamous carcinogenesis and angiogenesis.^59^ In MDA‐MB‐231 cells, the JNK, S6K and p38 MAPK pathways are responsive to PTX3, but this occurs through CD44‐independent regulation. Studies have demonstrated FcγR‐mediated activation of the JNK, S6K and p38 MAPK pathways in myeloid cells.^60^ However, issues such as whether and how FcγR is involved in the PTX3‐exerted protumour effect remain to be clarified.

Several studies support the idea that the glycosylation status of a protein can affect or change its cell signal transduction.^61^, ^62^, ^63^ Only one N‐glycosylation site was identified in PTX3; this is on the C‐terminus at Asn220.^20^ The site is primarily comprised of fucosylated and sialylated biantennary sugars with tri‐ and tetra‐antennary glycans.^20^ In the current study, the PTX3 and PTX3/C proteins containing glycosylation showed opposite tumourigenic effects from that of PTX3NA (Figure S6A–D). Enzyme‐linked immunosorbent assay (ELISA) revealed that both PTX3 and PTX3NA bind to CD44 with similar binding activity, indicating that the glycosylation status of PTX3 has no effect on binding with CD44. However, the synthetic PTX3 peptide inhibitors RI37 and AD9 and the PTX3NA protein dramatically inhibited PTX3‐induced signalling and downstream gene activation and inhibited the protumour effects of sphere formation and migration/invasion in breast cancer. These results indicate that glycosylation of PTX3 plays a critical role in PTX3‐involved regulation and its consequent protumour activity. In addition, the region of the GI40 peptide was also predicted on the surface of PTX3 and exerts antitumour activity. Co‐treatment with P2rdAD9 and P12rdGR9 showed an additive effect on the inhibition of breast cancer cell migration. Therefore, the GR9 region of PTX3 should have a collaboratory function to support or benefit the conformational change in the PTX3/CD44 interaction. The C‐terminus of PTX3 contains a homologous pentraxin domain similar to PTX family members, including CRP and SAP. However, AD9 and GR9 do not have similar sequences to the above PTX family members. These peptides specifically block the PTX3/CD44 signalling pathway; therefore, the off‐target effects caused by their high similarity to CRP and SAP are not of concern. Moreover, the PTX3 antibody also exhibited a specific interaction with PTX3 but not with CRP or SAP (Figure S12). Due to their ability to interfere with the PTX3–CD44 interaction, the PTX3 peptide P2rdAD9 and the PTX3 antibody Ab‐10 are good drug candidates and have the potential to be used for TNBC treatment. Although several CD44‐specific inhibitors have been developed over the past 10 years, a usable clinical drug specifically targeting CD44 is still lacking.^17^ This may result from the missing information of targetable and druggable CD44‐interacting proteins. Here, regarding the discovery of PTX3 and CD44 interactions, specific PTX3 inhibitors open a new avenue for further application in all PTX3/CD44‐specific pathogenic diseases.

No efficient targeted therapy is currently available for the treatment of TNBC. Therefore, chemotherapy remains the standard strategy for TNBC patients.^64^ However, these patients often show unsatisfactory responses to current treatments,^65^, ^66^ and there is a high recurrence rate within 1–3 years.^67^, ^68^ Increasing evidence supports the contribution of the TME in tumour malignancy and has dissected the details regarding its role. In addition, the genetic background of cancer cells, including TNBC, is heterogeneous compared to that of the surrounding cells. Therefore, identifying and focusing on critical protumour factors from tumour‐associated stromal cells could open new avenues for cancer therapies. We previously demonstrated that stromal PTX3 production induced immunosuppression and chemotherapy resistance.^25^, ^30^ Here, we further demonstrated that PTX3 is associated with high metastasis of primary breast cancers and poor prognosis. In addition, suggesting that PTX3 may represent a cancer biomarker of breast cancer progression. TNBC patients can be distinguished by high and low PTX3 population for the administration of PTX3 inhibitors. By survey from literature, no obvious syndrome was observed except to a pregnancy problem in PTX3‐deficient mice.^56^ However, the limitations including effects on immune responses need more investigation. Actually, the results of pharmacological toxicity and safety assays indicated that no survival, food consumption, body weight, clinical signs, clinical pathology and cardiovascular measurements were observed on endpoints while administrating humanised PTX3 antibody on primates. Our current study proposes targeting PTX3 as a treatment for TNBC patients.

## 4 MATERIALS AND METHODS

### 4.1 Cell culture and treatment

Human breast cancer MDA‐MB‐231 cells and mouse breast cancer 4T1 cells were acquired from the Bioresource Collection and Research Center (BCRC). BCAFs, were kindly provided by Dr. Kelvin K. Tsai. Dulbecco's modified Eagle's media (DMEM) were used to maintain these cells. Mouse breast cancer E0771 cells were purchased from *CH3* BioSystems and incubated in Roswell Park Memorial Institute. All culture media were added with 10% foetal bovine serum, streptomycin (100 mg/ml) and penicillin (100 U/ml).

### 4.2 Mouse models

For the animal model, MB‐Luc2 cells were mixed with human BCAFs xenografts: 1 × 10^5^ MB‐Luc2 were mixed with either 1 × 10^5^ BCAFs carrying a control knockdown vector (shCtl‐BCAFs), or 1 × 10^5^ BCAFs carrying a CEBPD knockdown vector (shCD‐BCAFs), or 1 × 10^5^ BCAFs carrying a PTX3 knockdown vector (shP‐BCAFs). The cell mixtures were inoculated into the mammary fat pads of NOD‐SCID mice. In peptide treatment experiments, NOD‐SCID mice were orthotopically inoculated with 1 × 10^6^ mCherry fluorescent‐expressing MDA‐MB‐231 cells into the mammary fat pad. Similarly, 5 × 10^5^ mCherry fluorescent‐expressing 4T1 cells were orthotopically inoculated into BALB/c mice in the mammary fat pad. Once tumours reached to the mean of 50 mm^3^, the mice were administered 40 mg/kg of peptides RI37, GI40, P2rdAD9 or P12rdGR9 every day by intraperitoneal injection. For paclitaxel treatment, 30 mg/kg of paclitaxel was administered to the experimental mice once a week. In the antibody treatment experiments, 1 × 10^6^ MB‐Luc2 were orthotopically inoculated into NOD‐SCID mice in the mammary fat pad. Similarly, 5 × 10^5^ 4T1‐Luc2 were orthotopically inoculated into BALB/c mice in the mammary fat pad. Once tumours measure up the mean of 50 mm^3^, 10 mg/kg of PTX3 antibody (PTX3 Ab‐10 or PTX3 Ab‐49), 10 mg/kg isotype antibody (IgG1κ) (with or without 30 mg/kg of paclitaxel) was administered to the experimental mice once a week. In *Cebpd*‐knockout mice and tamoxifen‐induced conditional *Ptx3‐*deficient mice, 5 × 10^5^ E0771‐Luc2 were orthotopically inoculated into *Cebpd*‐deficient mice, *ptx3^fl/fl^; UBC‐Cre* mice or Ptx3*^fl/fl^* mice in the mammary fat pad. The images of in vivo bioluminescence and metastasis quantification of the lung and brain were analysed by a Xenogen IVISR Spectrum Noninvasive Quantitative Molecular Imaging System. External calipers were then used to determine the size of tumour. After that, tumour volume was mathematically determined by using the standard formula: *V* = (*w* × *l* ^2^) × 0.52, where *l* represents the length and *w* represents the width of the tumour. The experimental animals were immolated at predetermined times after tumour cell inoculation. After, the number of nodules on the lung surface was measured. Experiments were followed by guidelines form Laboratory Animal Center, Medical college, National Cheng Kung University. In this study, all the animal use protocols were approved by the Institutional Animal Care and Use Committee (IACUC).

### 4.3 Establishment of gene knockout and transgenic mice

Six‐week‐old female C57BL/6 mice were acquired from the National Laboratory Animal Center (NLAC). *Cebpd*‐deficient mice with C57BL/6 strain were from E. Sterneck (National Cancer Institute, Frederick, MD, USA). The Ptx3*^fl/fl^* mice were bred in the Transgenic Mouse Model Core (Taiwan) by flanking loxP into exon 2 and 3 of Ptx3 using the CRISPR/Cas9 endonuclease method. Crossing *Ubc‐Cre‐ER* ^T2^ transgenic mice (Jackson Laboratories) with Ptx3*^fl/fl^* mice generated *Ptx3 ^fl/fl^*; *UBC*‐Cre mice (on a C57BL/6 background). The loxP recombination sites were analysed by polymerase chain reaction (PCR) using primers spanning exons 2 and 3. The following were the pairs of 5′ loxp primer—PTX3 5VF1 10446 (forward): 5′‐ ACTATCTGAGAAAAGCCAGAGGTTT‐3′ and PTX3 5VR1 10798 (reverse): 5′‐AGCATGATGAACAGCTTGTCCC‐3′, 3′ loxp primer—PTX3 3VF2 16115 (forward): 5′‐TTCAACTGAGGAGCAGAGGA‐3′ and PTX3 3VR2 16524 (reverse): 5′‐ACTTTGTGTGTCCCGGTACA‐3′.

The agarose gel electrophoresis was stained with SYBR™ Safe DNA Gel Stain (S33102; Thermo Fisher Scientific) for analysis of PCR products. Recombination between LoxP sites catalysed by Cre recombinase was induced by tamoxifen injection on day 3. For Cre activation, tamoxifen (Sigma‐Aldrich) was dissolved in corn oil at 20 mg/ml, first and then intraperitoneally injected into *Ptx3^fl/fl^*; *UBC*‐Cre or Ptx3*^fl/fl^* mice once per day for 5 consecutive days to systematically delete the floxed gene.

### 4.4 Patients and human materials

The formalin‐fixed tissue of patients and clinical database for this study were approved by the Chi‐Mei Medical Center (IRB10210004). Between 1997 and 2002, the immune expression of 72 cases of primary TNBC undergoing continuous treatment with modified radical mastectomy was evaluated. These patients with adjuvant chemotherapy were exclude. Pathological staging was classified on the basis of the seventh edition of the AJCC Cancer Staging Manual. To further evaluate the expression statuses of CEBPD and PTX3 in relation to chemoresistance, 20 cases receiving neoadjuvant chemotherapy were included and their pre‐ and post‐therapeutic tumour specimens were tested by means of immunohistochemistry (IHC).

### 4.5 Immunohistochemistry and scoring

The immunohistochemical studies were executed as mentioned before.^69^ The tissue slides were prepared with specific antibodies targeting CEBPD (sc‐636; Santa Cruz Biotechnology) and PTX3 (ab90806; Abcam) in human samples. Antibodies signal were scouted by exploiting the ChemMate DAKO EnVision kit (K5001; DAKO). Immunoexpression was rated by a professional pathologist (Chien‐Feng Li). The positively stained tumour cells were evaluated according to the percentage and intensity of to summarise an H‐score.^69^

### 4.6 Statistical analysis

The SPSS 14 software package was used for statistical analysis. The Mann–Whitney *U*‐test and chi‐square evaluation of the differential expression levels of CEBPD and PTX3 expression were related to important clinicopathological variables. The end points of the analysis were distal MeFS and DSS, counting from the date of surgery to the date of disease‐related death or metastasis events. The cox proportional hazards model was used for univariate survival analysis. The survival curve was analysed by Kaplan–Meier method. Log‐rank test was assessed the prognostic difference of categorical variables between groups. Multivariate model analysis was carried out by Cox proportional hazard regression, including univariate *p* < .05 parameters. Because primary tumour status (pT) and lymph node status (pN) are components of tumour staging, multivariate analysis only includes this staging. For all analyses, using a two‐sided significance test, *p* ≤ .05 is considered statistically significant.

### 4.7 Short hairpin RNA assay

Ampho Phoenix were used to produce lentiviruses by co‐transfecting with shRNA expression vectors or expression vectors. After ascertaining the viral infection efficiency, cells were infected with control (shCtl), shCEBPD (shCD), shPTX3 (shP) or shCD44 of lentiviral‐mediated gene knockdown for 48 h. The shRNA sequences which were subcloned into the lentiviral‐expressing vectors. Sequences were as follows: shCtl, 5′‐CCGGAGTTCAGTTACGATATCATGTCTCGAGACATTCGCGAGTAACTGAACTTTTTT‐3′; shCD, 5′‐CCGGGCTGTCGGCTGAGAACGAGAACTCGAGTTCTCGTTCTCAGCCGACAGCTTTTT‐3′; shP, 5′‐ CCGGGAGGAGCTCAGTATGTTTCATCTCGAGATGAAACATACTGAGCTCCTCTTTTTTG‐3′; shCD44, 5′‐ CCGGCCGTTGGAAACATAACCATTACTCGAGTAATGGTTATGTTTCCAACGGTTTTTG‐3′. The lentiviral knockdown expression vectors were purchased from National RNAi Core Facility, Taiwan.

### 4.8 Western blot

Experimental cells were lysed with radioimmunoprecipitation assay buffer (RIPA) buffer. Specific antibodies including PTX3 (ab90806; Abcam), CEBPD (sc‐636; Santa Cruz Biotechnology), CD44 (GTX83114; GeneTex), α‐tubulin (T6199; Sigma), P84 (GTX70220; GeneTex), RAC1 (GTX100761; GeneTex), CDC42 (GTX100904; GeneTex), AKT (GTX121937; GeneTex), Phospho‐AKT (Ser473) (GTX128414; GeneTex), Phospho‐NF‐κB p65 (Ser536) (#93H1; Cell Signaling), RhoA (ab68826; Abcam), NF‐κB p65 (#3033; Cell Signaling), Phospho‐p38 MAPK (Thr180/Tyr182) (#9211; Cell Signaling), PTEN (ab32199; Abcam), p38 MAPK (#9212; Cell Signaling), Phospho‐SAPK/JNK (Thr183/Tyr185) (#4668; Cell Signaling), Phospho‐p70 S6 Kinase (Thr389) (MABS82; Millipore), Phospho‐PAK1 (Ser199/Ser204) (09‐258; Millipore), SAPK/JNK (#9252; Cell Signaling), Phospho‐p44/42 MAPK (ERK1/2) (Thr202/Tyr204) (#4377; Cell Signaling) and p44/42 MAPK (ERK1/2) (#9102; Cell Signaling) were used for Western blotting.

### 4.9 In vivo DNA binding assay

The in vivo DNA binding assay was executed as bewrited by Wang et al.^70^ The experimental BCAFs were fixed with 1% formaldehyde after treating with 5 ng/ml of TGF‐β1 for 24 h. Afterward, sonicated the cross‐linked chromatin to 500 bp. The DNA fragments were then immunoprecipitated with control immunoglobulin G (IgG) or CEBPD antibodies. Then, the immunoprecipitated chromatin was enlarged by using specific primers to target the PTX3 genomic locus. The pair of PTX3 primers: 5′‐GCTCGGATTGGACTTGACTT‐3′ and 5′‐GAGGGAAATGTGGAAGTTGC‐3′. The enlarged DNA were then separated by agarose gel electrophoresis.

### 4.10 Reporter plasmids and luciferase assay

The PTX3 promoter was cut off by HindIII and KpnI from pGL2‐basic/PTX3^27^ and subcloned to the pGL3‐basic vector. *PTX3* reporter and expression vectors transfected to cells by using transfection reagent TransIT‐2020 (Mirus) for 18 h. Then, cells treated with 5 ng/ml of TGF‐β1 for 24 h. Next, cell lysates were harvested to conduct the luciferase assay.

### 4.11 Real‐time quantitative PCR assay

TRIsure RNA extraction reagent (Invitrogen) extracted total RNA. SuperScript™ III Reverse Transcriptase (Invitrogen) was used to synthesis cDNA. Next, SensiFAST™ SYBR (BIOLINE) was applied to perform real‐time quantitative PCR (Q‐PCR) assay. CFX connect Real‐Time PCR System (BIO‐RAD) executed real‐time fluorescence monitoring and the melting curve analysis according to the manufacturer's recommendations. The following pairs of specific primers—human CEBPD (F): 5′‐GCCATGTACGACGACGAGAG‐3′ and CEBPD (R): 5′‐TGTGATTGCTGTTGAAGAGGTC‐3′, human PTX3 (F): 5′‐ ATAGTGTTTGTGGTGGGTGGA‐3′ and PTX3 (R): 5′‐ ATGTGAGCCCTTCCTCTGAAT‐3′, human MMP2 (F): 5′‐ CCCTGTGTCTTCCCCTTCAC‐3′ and MMP2 (R): 5′‐ GTGGTCGCACACCACATCTT‐3′, human RANKL (F): 5′‐ AGAGC GCAGATGGATCCTAA‐3′ and RANKL (R): 5′‐ TTCCTTTTGCACAGC TCCTT‐3′, human TWIST2 (F): 5′‐AGATCCAGACGCTCAAGCTG‐3′ and TWIST2 (R): 5′‐ATTGTCCATCTCGTCGCTCT‐3′, human NANOG (F): 5′‐TTCTTACCGTTTTTGGCTCTG‐3′ and NANOG (R): 5′‐ GGAAAAATAGTTGTTTCATTCATTTG‐3′, human GAPDH (F): 5′‐ CCATCACCATCTTCCAGGAG‐3′ and GAPDH (R): 5′‐ CCTGCTTCACCACCTTCTTG‐3′. The result of each group was normalised with GAPDH, respectively, and was showed as the difference in folds.

### 4.12 Migration and Matrigel invasion assays

In migration assay, MDA‐MB‐231 cells seeded in 8.0 μm pore polycarbonate membrane inserts (BD Biosciences) in 24‐well plates. In Matrigel invasion assay, inserts were pre‐coated with an appropriate amount of Matrigel first, and the same number of cells seeded on the Matrigel. After cells adhered on the inserts, serum‐free DMEM medium added in the upper wells. Serum‐free medium containing the treatments of TGF‐β1, PTX3, PTX3NA, PTX3/N, PTX3/C, RI37, GI40, AD9, GR9, IgG1κ, PTX3 Ab‐10 or PTX3 Ab‐49 were then mixed into the lower wells of the 24‐well plates. After incubating for 18 h, cells outside the insert were removed by wiping. The inside the inserts were then stained by 4′,6‐diamidino‐2‐phenylindol (DAPI). Fluorescence microscope was used for counting the total number of migrated cells with 200× magnification. Different from the experimental design for the MDA‐MB‐231 cells, the migrated 4T1‐Luc2 cells in the inside of the insert were detected by luciferase activity.

### 4.13 Sphere formation assay

Note that 5 × 10^3^ MDA‐MB‐231 cells were seeded in ultralow attachment plates (Corning) per well. Then, cultured in conditional medium containing the treatment of TGF‐β1, PTX3, PTX3NA, RI37, GI40, IgG1κ, PTX3 Ab‐10 or PTX3 Ab‐49 and the DMEM/F12 (Gibco) added with B27 (Invitrogen), 10 ng/ml bFGF (Peprotech) and 20 ng/ml EGF (Abcam). Number of spheres was calculated by microscope after 2 weeks.

### 4.14 Co‐immunoprecipitation assay

The membrane protein of MDA‐MB‐231 cell was extracted by Mem‐PER™ Plus kit (Thermo) following the manufacturer's steps. Collected the membrane protein and incubate with His‐PTX3, and then incubated with the specific antibody that recognises PTX3 (ab90806; Abcam) or CD44 (GTX83114; GeneTex) at 4°C overnight. After, agarose beads of protein‐A/G were supplemented into the lysates, and mixtures were centrifuged to collect the beads, and the beads were washed by RIPA buffer for three times. To elute the proteins which were bound to the beads by using electrophoresis buffer to combine with beads, and then perform by Western blot.

### 4.15 Surface plasmon resonance analysis

The BIAcore 3000 system (GE Healthcare Co. Ltd.) was used to determine the direct protein interaction between the full‐length PTX3 (#1826‐TS; R&D Systems) and the N‐terminal of CD44 (#CD44‐3961H; Creative Biomart). Full‐length PTX3 was immobilised onto a CM5 sensor chip (GE Healthcare Co. Ltd.); the immobilisation level was 4000–6000 response units using amine‐coupling chemistry. Briefly, a mixture of 1‐Ethyl‐3‐(3‐dimethylaminopropyl) carbodiimide hydrochloride (EDC) and *N*‐hydroxysuccinimide activated the surface of the chip, and then diluted full‐length PTX3 (10 μg/ml in acetate buffer pH 3) was injected into the flow cells for immobilisation. Finally, the remaining activated groups were blocked by ethanolamine. The binding parameters of the experiment were measured over a 1‐min association phase and then followed by a 2‐min dissociation phase at a fluid flow rate of 10 μl/min. Phosphate‐buffered saline (PBS, pH 7.2) was used as the analyte running buffer; 0.05% sodium dodecyl sulphate was used to regenerate the chip surface before the next analyte concentration was applied. The 1:1 Langmuir binding model (BIAevaluation 4.1; GE Healthcare Co. Ltd.) were performed to calculate its corresponding association and dissociation rate constants to determine the affinity.

### 4.16 Immunofluorescence, confocal microscopy and image analysis

MDA‐MB‐231 treated with His‐PTX3 fusion protein for 24 h. Four percent paraformaldehyde was used to fix the cells for 20 min. Cells were then incubated with antibody against CD44 (GTX83114; GeneTex) or His (sc‐803; Santa Cruz Biotechnology). In immunofluorescence, the samples were stained with Alexa488‐ or 568‐conjugated secondary antibodies (Invitrogen). The fluorophores were excited by a laser at 405, 488 or 543 nm and were observed using the FV‐1000 confocal system (Olympus).

### 4.17 Proximity ligation assay

MDA‐MB‐231 cells seeded on coverslips then treated with or without His‐PTX3 fusion protein for 1 h. Before fixing with 4% paraformaldehyde, the cell membrane was stained with PKH67, then PLA according to manufacturer's instructions (Sigma). Two primary antibodies were used to recognise His‐PTX3 and the active form of CD44. The secondary antibodies are PLA probes. The protein interactions are magnified into different bright red spots and evaluated by fluorescence microscopy. The randomly selected fields of view were photographed at a magnification of ×1000. Quantify protein interactions by calculating the number of spots per cell.

### 4.18 Microwestern array analysis

The MDA‐MB‐231 and CD44 receptor knockdown MDA‐MB‐231 cells were incubated with 200 ng/ml PTX3 for 30 min before being harvested. Microwestern array was applied to detect the levels of protein and phosphorylated protein. β‐Actin and α‐tubulin were utilised as loading controls. Scanned images were acquired by Odyssey Infrared Imaging System. The intensity of the protein bands was estimated by using Odyssey 3.0 software. One hundred and ninety‐two antibodies related to various signalling pathways were selected and used to perform the microwestern array using previously described methods.^71^

### 4.19 Peptide stability, ligand binding and competitive binding assays

Plate wells (9018; Corning Costar) were coated with 10 μg/ml of CD44 (12211‐H08H; Sino Biological Inc.) in PBS (pH 7.2) and then blocking for 1 h with the blocking buffer including 3% milk in PBS. For the stability binding experiments, the PTX3‐devired biotinylated peptides were pre‐incubated in serum for 1, 2, 4 or 6 h. For the binding experiments, the horseradish peroxidase (HRP)‐conjugated recombinant PTX3, PTX3/N, PTX3/C and PTX3NA or PTX3‐devired biotinylated peptides AD9, GR9, P2rdAD9 and P12rdGR9 (32310; LEADGENE) was responded in the well for 2 h. For the peptide of competition experiments, the PTX3‐devired biotinylated peptides RI37 and GI40 were diluted at the indicated concentration in PBS. After 2 h, PBS washed three times and then incubated with 5 μg/ml HRP‐conjugated PTX3 in PBS for 1 h at room temperature (RT). For the antibody competition experiments, Ab‐10 and Ab‐49 were incubated with 5 μg/ml HRP‐conjugated PTX3 in PBS for 2 h at RT. After incubation, the mixtures was responded in the well for 1 h at RT and then PBS washed three times, tetramethyl benzidine (TMB) was used to detect the bound HRP conjugate. After 15 min, the peroxidase reaction was stopped by adding 0.1 M H_2_SO_4_. Next, ELISA plate reader was used to detect the optical densities at 450 nm. The data show the results of three independent experiments.

### 4.20 Epitope mapping

The 200 μg/ml of the synthetic peptides RI37, GI40, AD9 or GR9 were coated into Plate wells (Corning Costar) at 4°C overnight and then blocked for 1 h at RT (1% bovine serum albumin (BSA) in PBS). For the binding experiments, 125 ng/ml Ab‐10 or Ab‐49 was incubated for 2 h at RT. Then, washed three times with PBS and incubated with anti‐mouse IgG‐HRP (1:5000) for 1 h at RT. After PBS washed three times, the HRP was detected by TMB. After 10 min, the peroxidase reaction was stopped by adding 0.1 M H_2_SO_4_. Next, ELISA plate reader was used to detect the optical densities at 450 nm. The data show the results of three independent experiments.

### 4.21 Cell cytotoxicity assay

MTT (Sigma) were used to evaluate the viability of the 4T1 cells treated with anticancer drugs. The cancer cells were incubated with 200 ng/ml PTX3, 0.8 μg/ml of PTX3‐devired peptides P2rdAD9 or P12rdGR9, and with or without 50 nM paclitaxel (Taxol), 50 nM doxorubicin (Dox) or 30 μM cisplatin (CDDP) for 24 h.

## CONFLICT OF INTEREST

The authors declare no potential conflicts of interest.

## Supporting information

Supporting Information
