# Supplementary material for: Disruption of the pentraxin 3/CD44 interaction as an efficient therapy for triple‐negative breast cancers
Source: Clin Transl Med. 2022 Jan 28;12(1):e724. doi: 10.1002/ctm2.724 (PMC8797470; doi:10.1002/ctm2.724)
Supplement: Supplementary file 1 — Supporting Information [file CTM2-12-e724-s001.docx]

**Supplemental methods**

**Microarray**

The microarray experiment and data analysis were performed by Welgene Biotech Company (Taipei, Taiwan). 0.2 μg of total RNA was amplified by a Low Input Quick-Amp Labeling kit (Agilent Technologies, USA) and labeled with Cy3 (CyDye, Agilent Technologies, USA) during the in vitro transcription process. 0.6 μg of Cy3-labled cRNA was fragmented to an average size of about 50-100 nucleotides by incubation with fragmentation buffer at 60°C for 30 minutes. Correspondingly fragmented labeled cRNA is then pooled and hybridized to Agilent SurePrint Microarray (Agilent Technologies, USA) at 65°C for 17 h. After washing and drying by nitrogen gun blowing, microarrays are scanned with an Agilent microarray scanner (Agilent Technologies, USA) at 535 nm for Cy3. Scanned images are analyzed by Feature extraction10.5.1.1 software (Agilent Technologies, USA), an image analysis and normalization software is used to quantify signal and background intensity for each feature. Raw signal data was normalized by quantile normalization for differential expressed genes discovering. For functional assay, we provide enrichment test for differential expressed genes (For most model organisms). Welgene Biotech used clusterProfiler for enrichment test for gene ontology (GO) and pathway (KEGG).


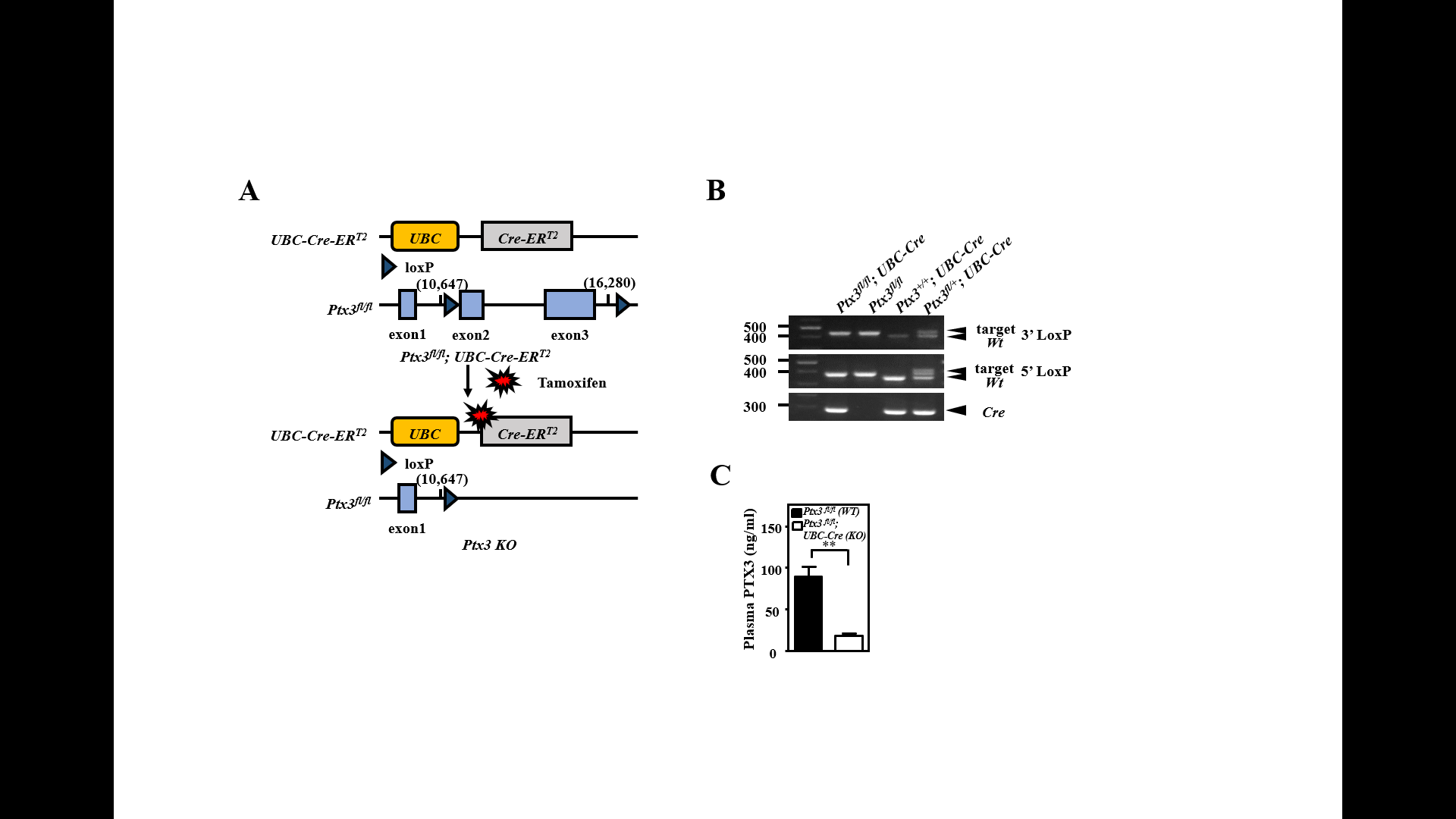


**Figure S1. Tamoxifen-induced *Ptx3*-knockout mice. (A)** The schematic representation of *Ptx3* KO mice model. Crossing *UBC-Cre-ER^T2^* transgenic mice with *Ptx3* *^fl/fl^* mice to generate *Ptx3 ^fl/fl^*; *UBC-Cre* mice. **(B)** Representative genotype PCR analysis of genomic DNA from tail tissues of *Ptx3^fl/fl^*; *UBC-Cre-ER^T2^*, in which “fl” represents floxed and “+” represents wild type. **(C)** The plasma PTX3 was measured via ELISA with plasma from E0771-Luc2-bearing *Ptx3* *^fl/fl^* (WT) or *Ptx3 ^fl/fl^*; *UBC-Cre* (KO) mice, as illustrated in **(A)**. All data are represented as the mean ± SEM. Differences between groups were analyzed with the unpaired two-tailed *t*-test. ***p* < 0.01.


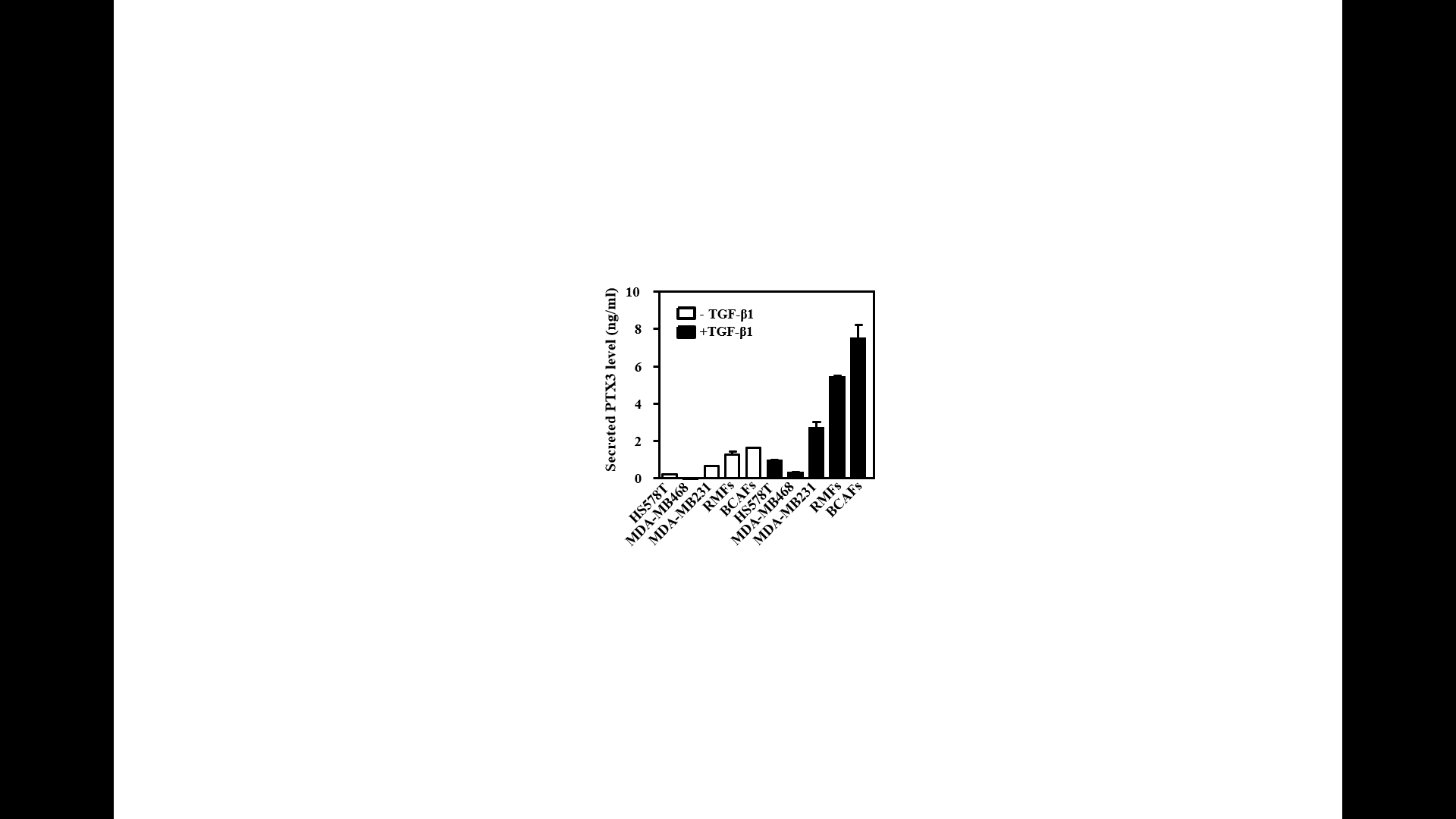


**Figure S2. PTX3 expression and response to TGF-β1 are significantly high in breast cancer-associated fibroblasts.** The supernatant was harvested from TGF-β-treated various breast cancer cells, breast fibroblasts (RMFs) and breast cancer-associated fibroblasts (BCAFs).


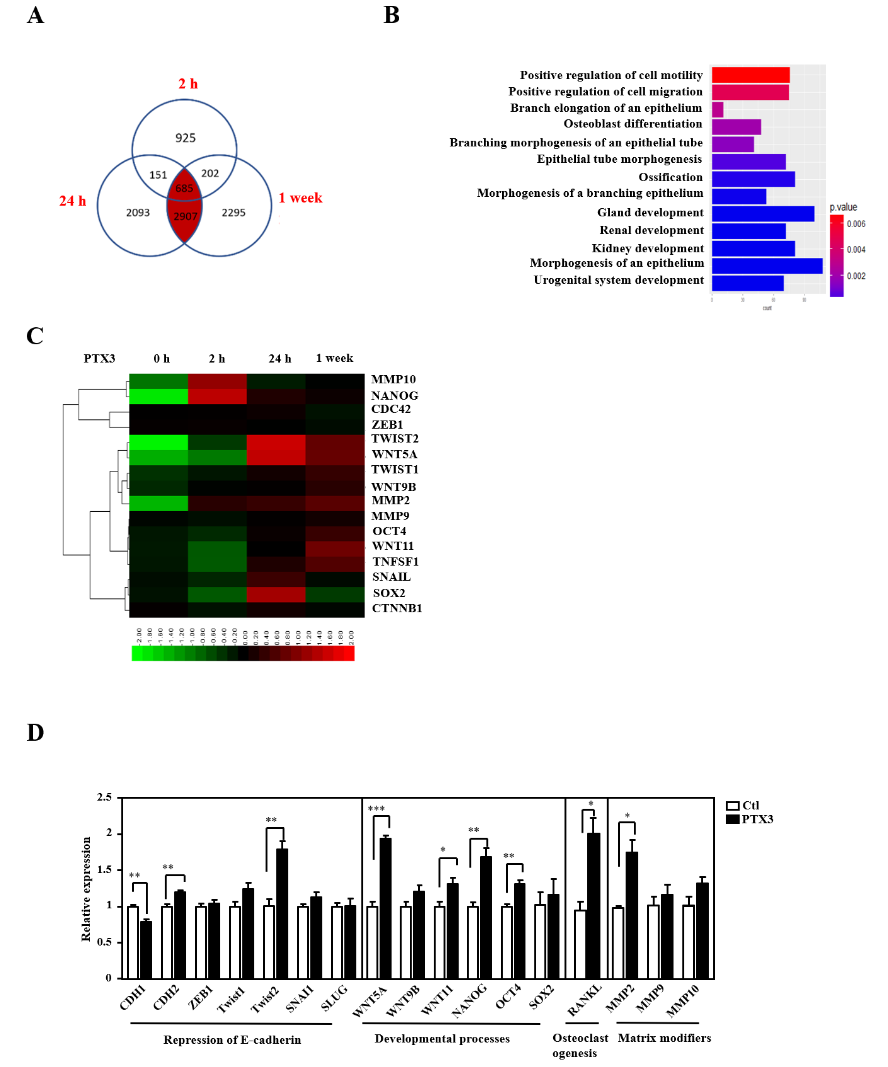


**Figure S3. Enrichment and validation analyses of genes exhibiting differential expression in PTX3-treated MDA-MB-231 cells.** **(A)** Venn diagram showing the overlap of changed genes in PTX3-treated MDA-MB-231 cells at 2 h, 24 h and 1 week. **(B)** Gene ontology (GO) biology process analysis was performed by mapping the overlap of changed genes shown in (A) using the clusterProfiler analysis tool. The GO terms of genes with a p value < 0.05 are shown here. **(C)** 16 most significantly upregulated genes with GO terms in PTX3-treated MDA-MB-231 cells and are represented as Heat map. **(D)** PTX3-responsive genes were verified in PTX3-treated MDA-MB-231 cells by real-time RT-PCR for 24 h of PTX3 treatment and represented by grouping of repression of E-cadherin, osteoclastogenesis, developmental processes and matrix modifier. All data are expressed as the mean ± SEM. Differences between groups were analyzed with the unpaired two-tailed *t*-test. *p < 0.05, **p < 0.01, ***p < 0.001.


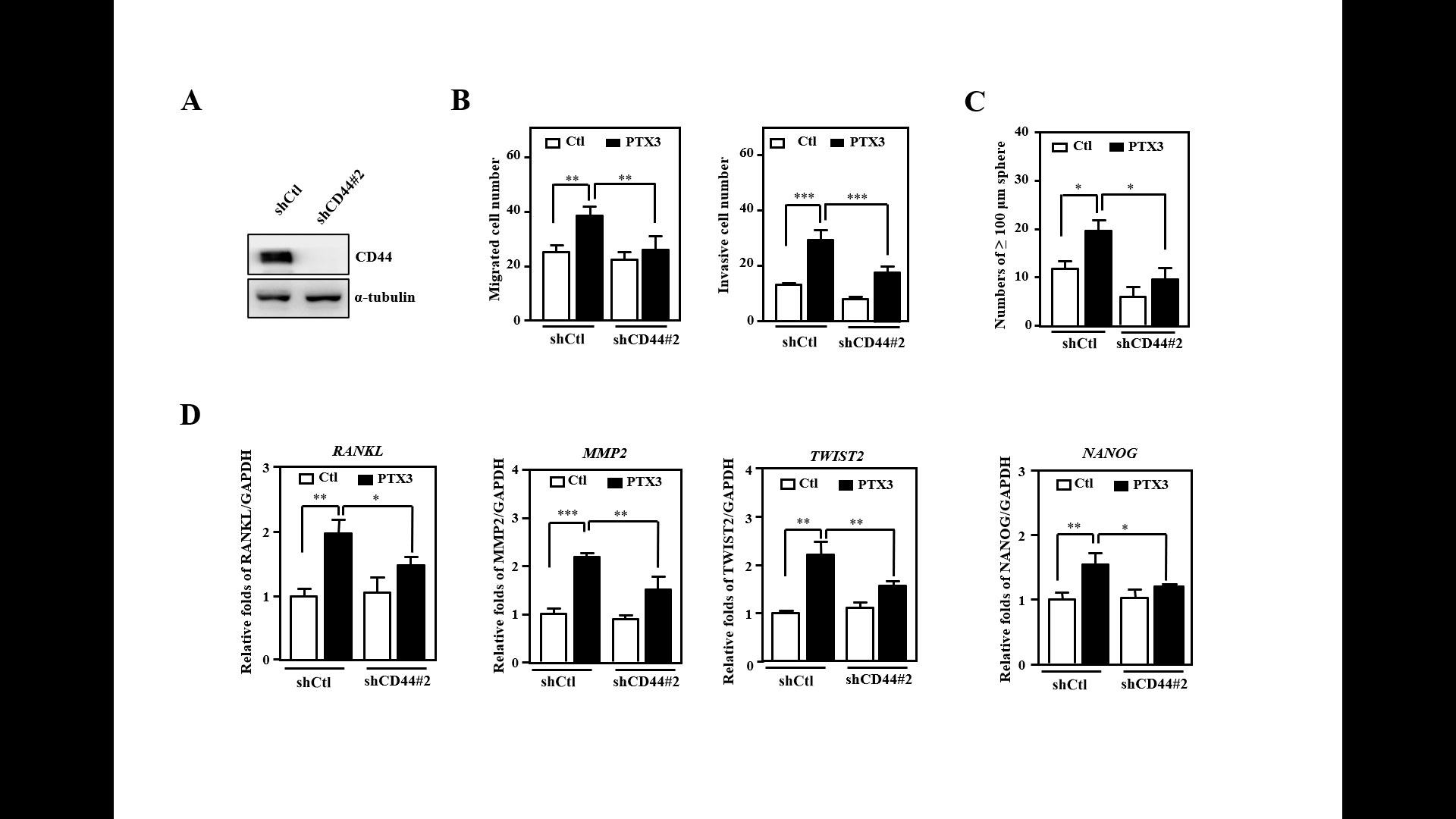


**Figure S4. PTX3 participates in migration, invasion, sphere formation and stemness gene expression** **via CD44 in MDA-MB-231 cells. (A)** MDA-MB-231 cells were infected with lentiviral control (shCtl) or shCD44#2. Lysates from experimental cells were harvested for western blotting. **(B)** Migration and invasion were assessed using Transwell assays with shCtl- or shCD44#2 MDA-MB-231 cells with or without PTX3 treatment. **(C)** The number of tumor spheres of shCtl or shCD44#2 MDA-MB-231 cells was counted after 7 days of incubation with or without PTX3 treatment. **(D)** MDA-MB-231 cells were preinfected with lentiviral shCtl or shCD44#2 and then treated with PTX3 for 24 h. The mRNA levels of *RANKL*, *MMP2*, *TWIST2* and *NANOG* were measured by real-time RT-PCR. *GAPDH* was used as an internal control. All data are expressed as the mean ± SEM. Differences among groups were analyzed with one-way ANOVA followed by Tukey’s multiple comparison test. *p < 0.05, **p < 0.01, ***p < 0.001.

**Figure S5.** **Quantitative analysis of the protein expression levels of CD44, CDC42, RHOA, RAC1, PTEN, p-PAK1, p-JNK, p-S6K, p-AKT, p-p65, p-ERK1/2 and p-p38 in MDA-MB-231 cells. (A)** Quantification of the relative protein level in MDA-MB-231 cells infected with lentiviral control (shCtl) or shCD44#1 and then treated in the presence or absence of PTX3. Relative protein expression was normalized to α-tubulin. **(B)** Quantification of the relative protein level in MDA-MB-231 cells pretreated with the indicated signaling inhibitors and then treated in the presence or absence of PTX3. p-AKT, p-p65 and p-ERK1/2 was normalized to the respective nonphosphorylated protein levels. All data are expressed as the mean ± SEM. Differences among groups were analyzed with one-way ANOVA followed by Tukey’s multiple comparison test. *p < 0.05, **p < 0.01, ***p < 0.001.


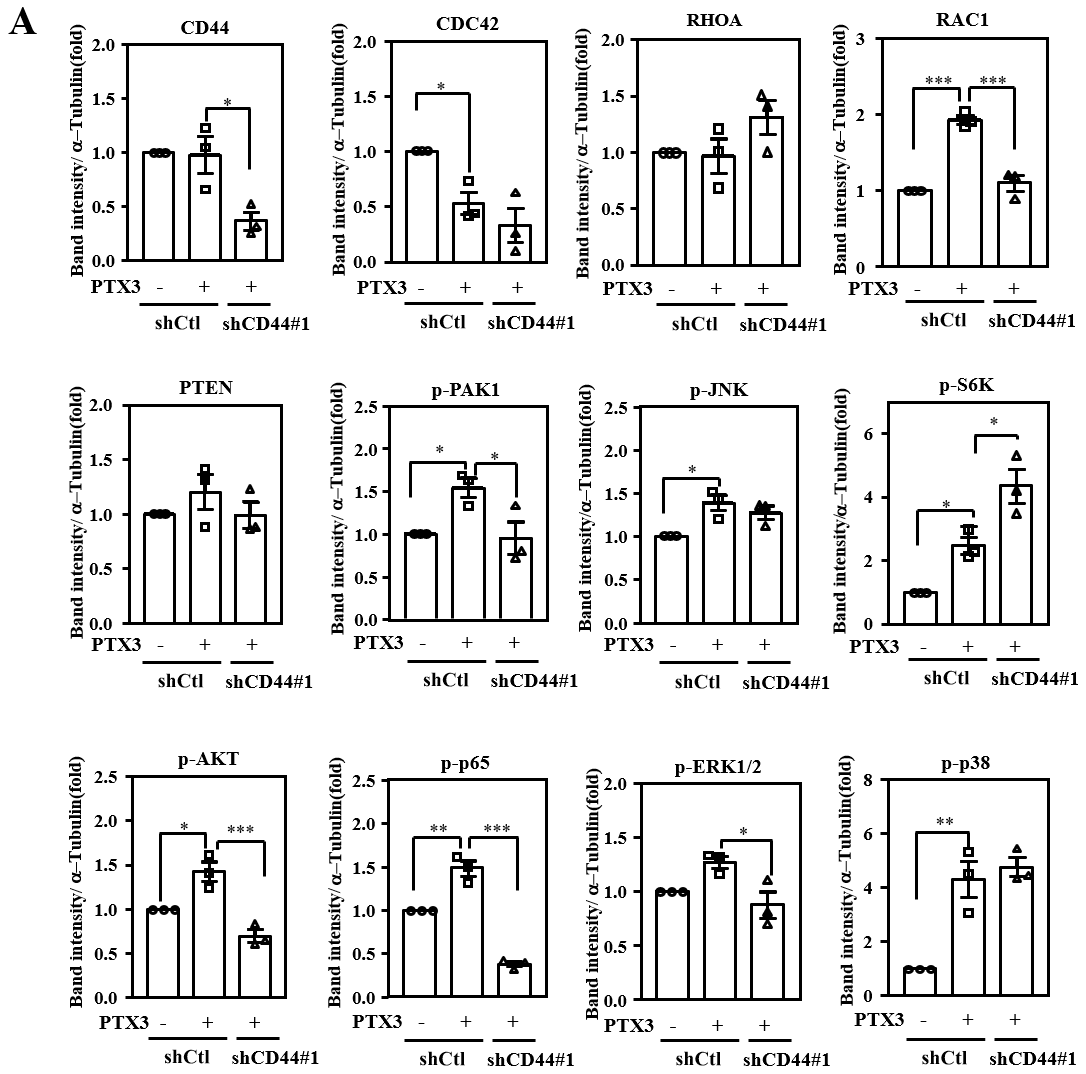


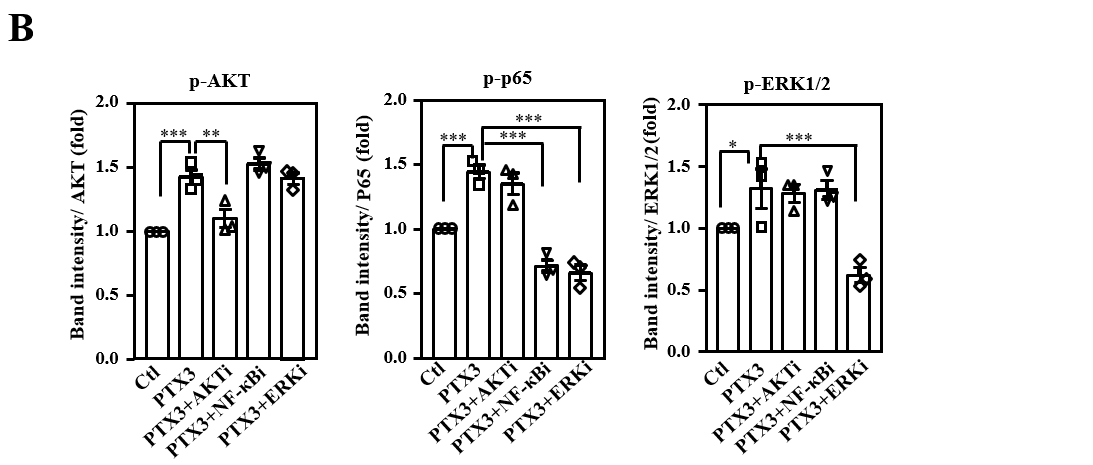


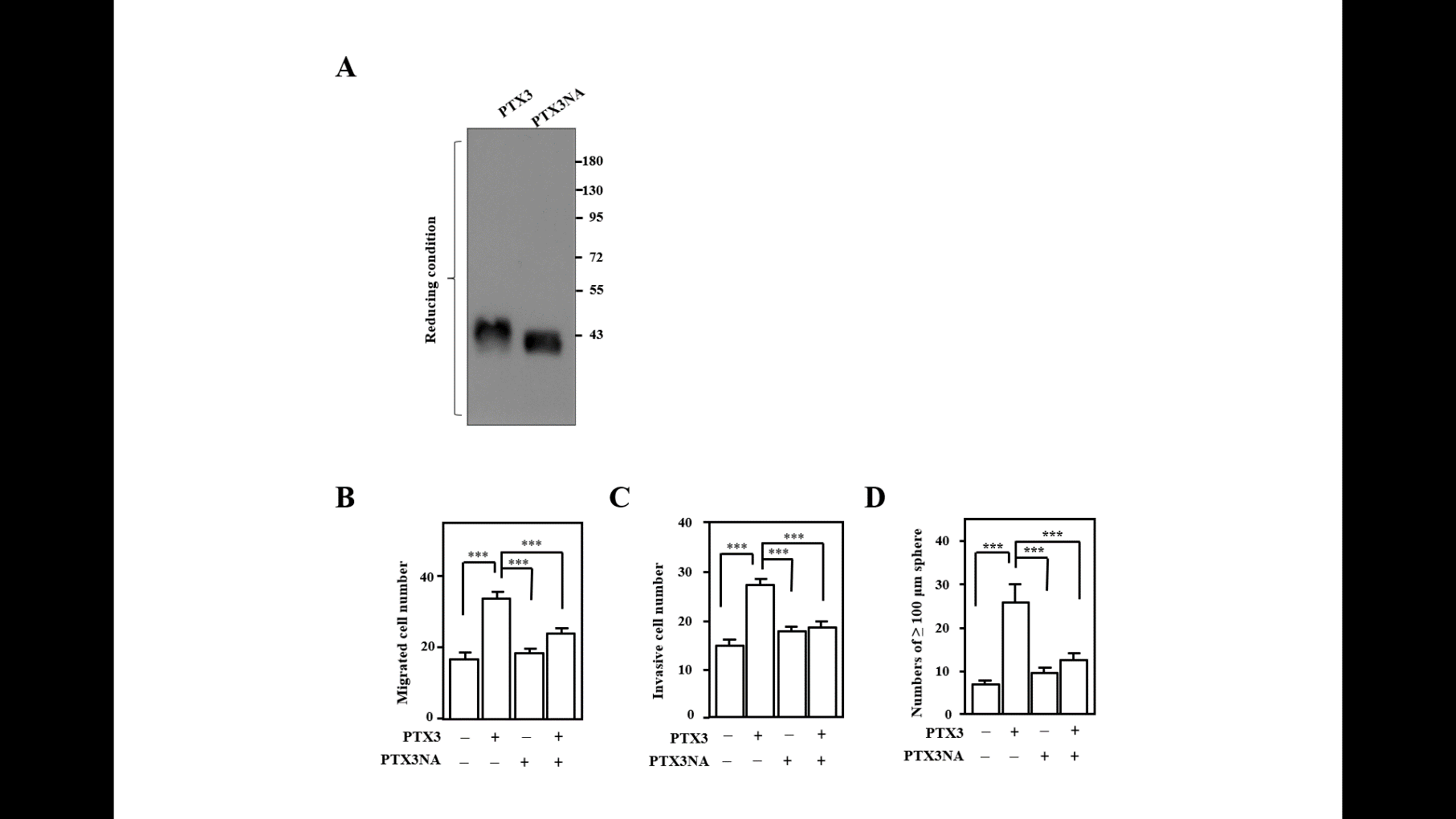


**Figure S6. Glycosylation mutant PTX3 attenuates PTX3-induced stemness and migration/invasion of breast cancer cells.** **(A)** Pattern of PTX3 and recombinant PTX3 Asn220 mutant PTX3 (PTX3NA) in a reducing gel. **(B)(C)** Transwell assay was performed to assess the migration and invasion of PTX3- and PTX3NA-treated MDA-MB-231 cells. **(D)** *In vitro* sphere formation assay was performed with PTX3- and PTX3NA-treated MDA-MB-231 cells. All data are represented as the mean ± SEM. Differences among groups were analyzed with one-way ANOVA followed by Tukey’s multiple comparison test. ***p < 0.001.

**
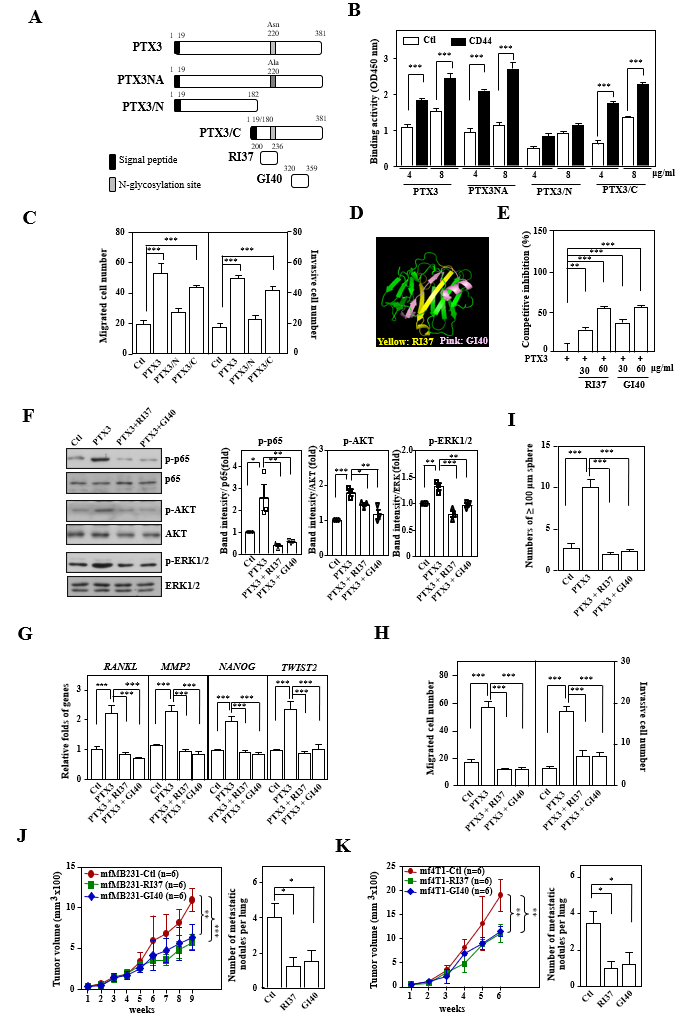
**

**Figure S7. PTX3 peptides compete for PTX3 binding to CD44 and inhibit the migration/invasion and stemness of TNBC. (A)** Schematic diagram of PTX3 truncation mutants and synthetic short PTX3 peptides. The position of the glycosylation site is labeled in gray (Asn) or dark gray (Ala). The signal peptide is labeled in black. The numbers indicate the positions of amino acids. **(B)** The binding ability of HRP-conjugated PTX3, PTX3/N, PTX3/C or PTX3NA with immobilized CD44 or gelatin (a negative control) was determined by ELISA. The samples were analyzed in quadruplicate. **(C)** A Transwell assay was performed with PTX3-, PTX3/C - or PTX3/C-treated MDA-MB-231 cells. **(D)** The structure of the PTX3 C-terminus was first remodeled with pentraxin family members SAP and CRP. Regarding the remodeling structure, the locations of RI37 (yellow, amino acids 200-236) and GI40 (pink, amino acids 320-359) were mapped onto the remodeled PTX3-C-terminus. **(E)** Competitive ability of RI37 and GI40 on PTX3 binding to CD44. The competitive binding assay was performed by ELISA. Samples were analyzed in quadruplicate. **(F)** Quantitative analysis of the levels of p-AKT, p-p65, and p-ERK1/2 in RI37- and GI40-treated MDA-MB-231 cells with or without PTX3 treatment and immunoblotted with the indicated antibodies. **(G)** Total RNA of RI37- or GI40-treated MDA-MB-231 cells with or without PTX3 treatment was prepared and analyzed using real-time RT-PCR with specific primers for the indicated genes. **(H)** The migration and invasion of RI37- and GI40-treated MDA-MB-231 cells with or without PTX3 treatment were assessed using a Transwell assay. **(I)** An *in vitro* sphere formation assay was performed with RI37- and GI40-treated MDA-MB-231 cells with or without PTX3 treatment. **(J)** The effects of RI37 and GI40 on the growth and metastasis of orthotopically xenografted mCherry fluorescent-expressing MDA-MB-231 cells (mfMB231) in NOD-SCID mice and **(K)** orthotopically allografted mCherry fluorescent-expressing 4T1 cells (mf4T1) in BALB/c mice**.** The injection of RI37 and GI40 was performed by I.P., and the experimental mice were sacrificed at the 9^th^ or 6^th^ week. The tumor volume was measured with external calipers. The metastasis of mfMB231-xenografted and mf4T1-allografted tumors to lung tissue nodules was calculated and is shown in their individual right panel. All data are represented as the mean ± SEM. Differences among groups were analyzed with one-way ANOVA followed by Tukey’s multiple comparison test. *p < 0.05, **p < 0.01, ***p < 0.001.

**
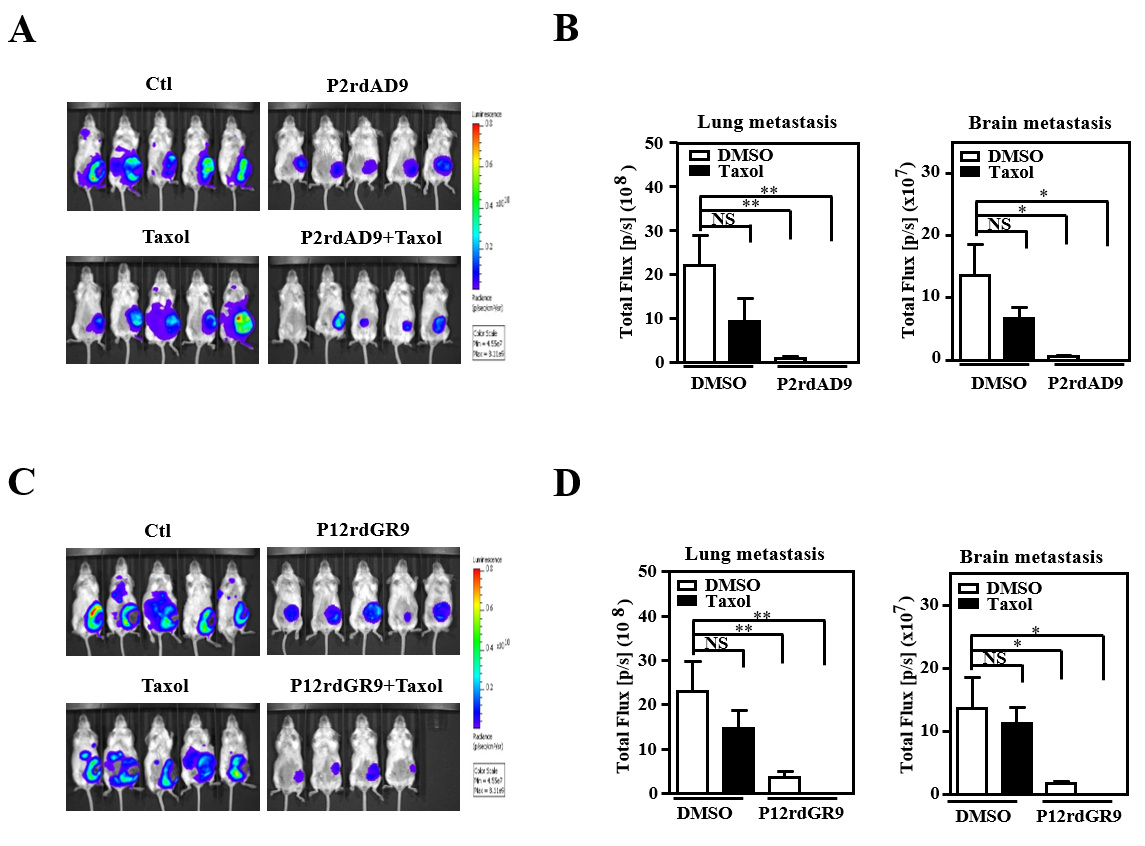
**

**Figure S8. The combination of PTX3 peptide** **and paclitaxel enhances the metastasis suppression in the TNBC mouse model. (A) (C)** Representative *in vivo* bioluminescence images and **(B) (D)** metastasis quantification of E0771-Luc2 tumors in lung and brain in biotinylated and PEGylated retro-inverso narrowed down PTX3 peptides AD9 (P2rdAD9)-, GR9 (P12rdGR9)-, or paclitaxel (Taxol)-treated 4T1-Luc2-bearing mice in the indicated groups at week 6. All data are expressed as the mean ± SEM. Differences among groups were analyzed with one-way ANOVA followed by Tukey’s multiple comparison test *p < 0.05, **p < 0.01, ns: no significance.

**
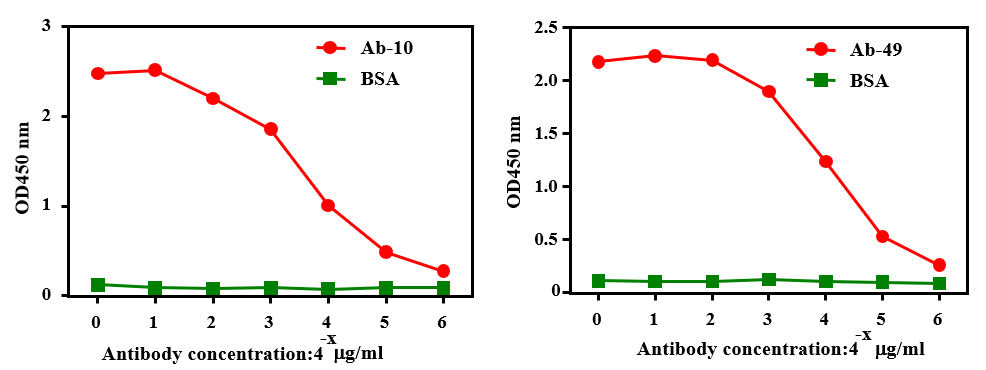
**

**Figure S9. PTX3 antibodies recognize PTX3 protein.** The ability of PTX3 antibodies (Ab-10 and Ab-49) bind to immobilized PTX3 or BSA. The binding assay was performed by ELISA and carried out in triplicate.


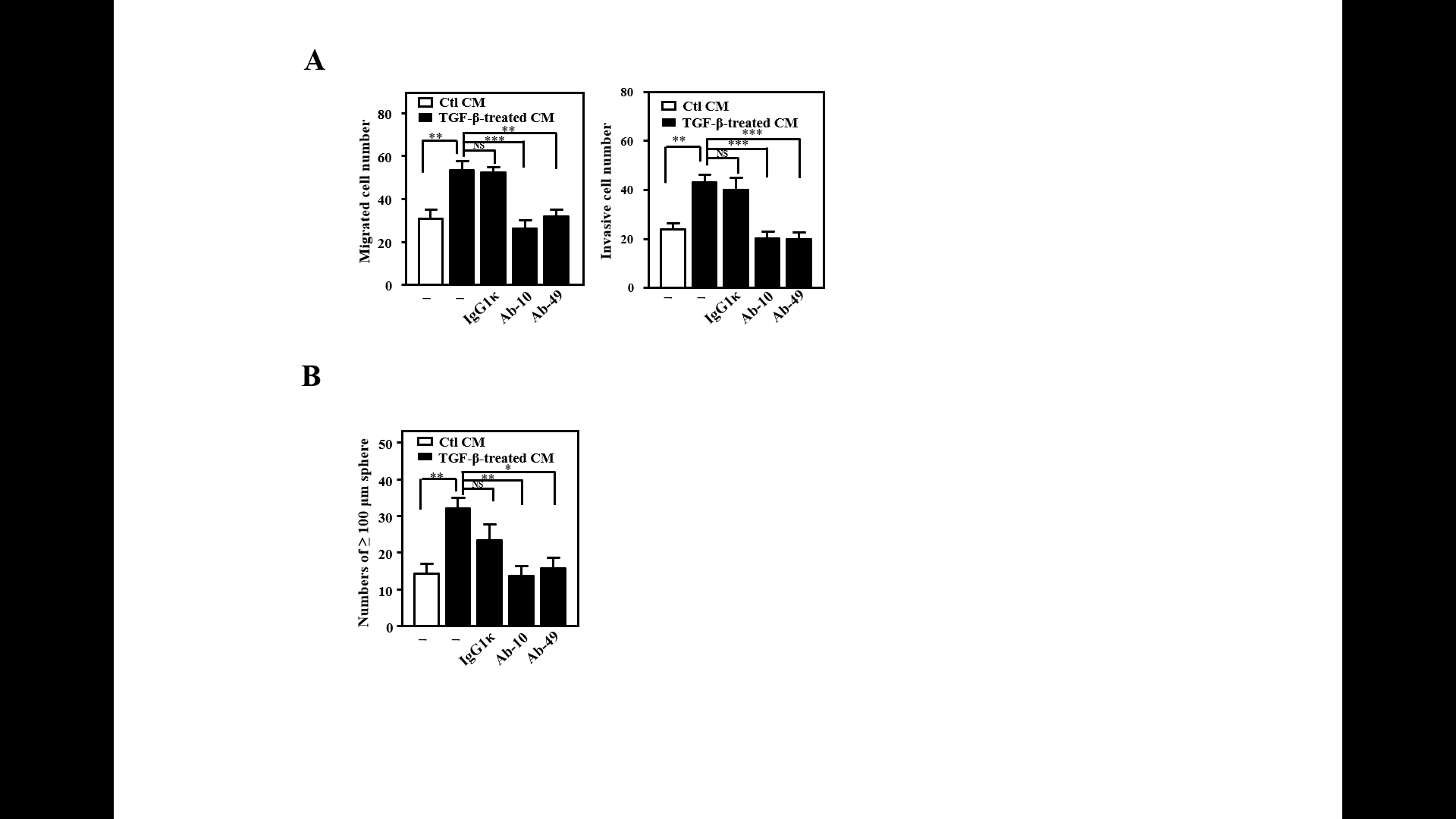


**Figure S10.** **Neutralized BCAFs PTX3 attenuates the migration, invasion and sphere formation of MDA-MB-231 cells. (A)** The migration and invasion of IgG1κ-, Ab-10- and Ab-49-treated MDA-MB-231 cells with or without conditioned medium from TGF-β-treated BCAFs were assessed by Transwell assay. **(B)** The sphere formation of IgG1κ-, Ab-10- and Ab-49-treated MDA-MB-231 cells with or without conditioned medium from TGF-β-treated BCAFs was assessed by sphere formation assay. Images of sphere formation of MDA-MB-231 cells were captured using phase contrast microscopy. All data are expressed as the mean ± SEM. Differences among groups were analyzed using one-way ANOVA followed by Tukey’s multiple comparison test. *p < 0.05, **p < 0.01, ***p < 0.001, ns: no significance.

**
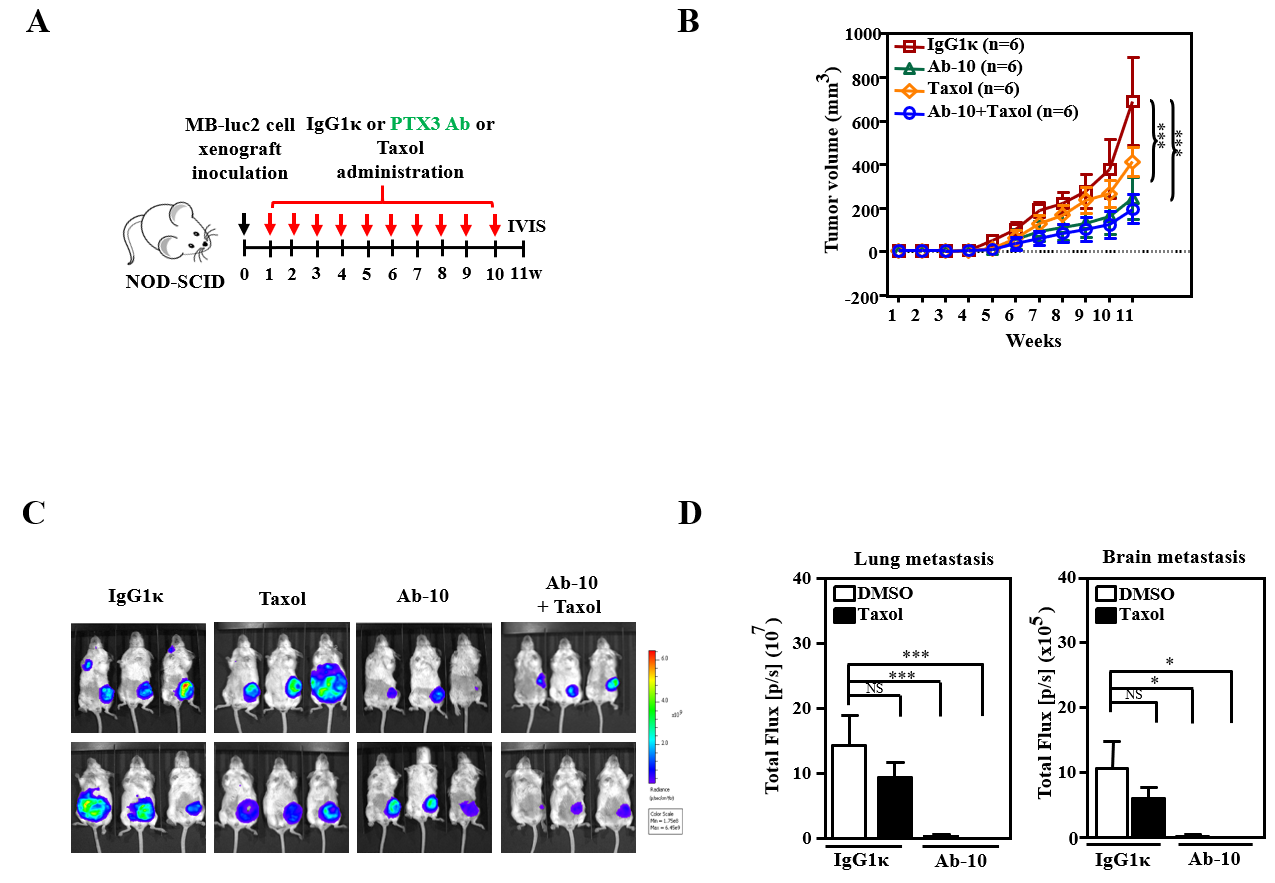
**

**Figure S11.** **PTX3 antibody suppresses tumor growth and metastasis in a TNBC mouse model. (A)** An experimental scheme for administering IgG1κ, PTX3 antibody (Ab-10) or paclitaxel (Taxol) in the indicated groups of orthotopically xenografted luciferase-expressing MDA-MB-231 cells (MB-Luc2) cell-bearing mice by intraperitoneal injection. **(B)** Tumor growth of MB-Luc2 cells in mice was measured with external calipers. **(C)** Representative *in vivo* bioluminescence images and **(D)** metastasis quantification of brain and lung in MB-Luc2-bearing mice in the indicated groups. All data are expressed as the mean ± SEM. Differences among groups were analyzed with one-way ANOVA followed by Tukey’s multiple comparison test *p < 0.05, ***p < 0.001, ns*:* no significance.


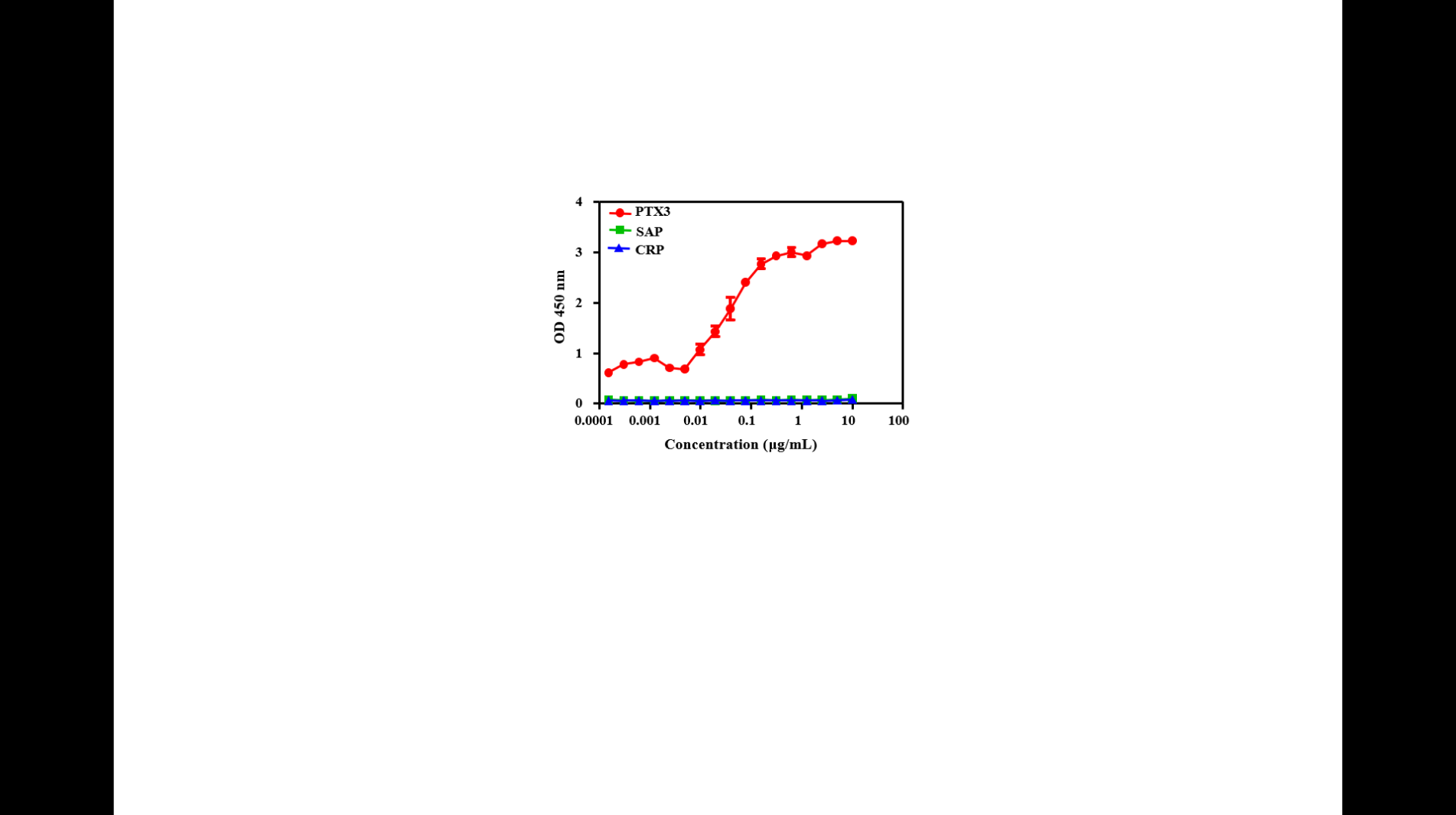


**Figure S12.** **PTX3 antibody specifically interacts with PTX3 but not CRP or SAP.** Recombinant PTX3, SAP and SAP were applied for assessing the cognition effect of Ab-10. The affinity was assessed by ELISA in triplicate.
